# Multi-omics Reveals the Lifestyle of the Acidophilic, Mineral-Oxidizing Model Species Leptospirillum ferriphilum^T^

**DOI:** 10.1128/AEM.02091-17

**Published:** 2018-01-17

**Authors:** Stephan Christel, Malte Herold, Sören Bellenberg, Mohamed El Hajjami, Antoine Buetti-Dinh, Igor V. Pivkin, Wolfgang Sand, Paul Wilmes, Ansgar Poetsch, Mark Dopson

**Affiliations:** a Centre for Ecology and Evolution in Microbial Model Systems, Linnaeus University, Kalmar, Sweden; b Luxembourg Centre for Systems Biomedicine, University of Luxembourg, Belvaux, Luxembourg; c Aquatic Biotechnology, Universität Duisburg-Essen, Essen, Germany; d Plant Biochemistry, Ruhr Universität Bochum, Bochum, Germany; e Institute of Computational Science, Faculty of Informatics, Università della Svizzera Italiana, Lugano, Switzerland; f Swiss Institute of Bioinformatics, Lausanne, Switzerland; g College of Environmental Science and Engineering, Donghua University, Shanghai, People's Republic of China; h Mining Academy and Technical University Freiberg, Freiberg, Germany; i School of Biomedical and Healthcare Sciences, Plymouth University, Plymouth, United Kingdom; University of Buenos Aires

**Keywords:** *Leptospirillum*, acidophile, biomining, genome, metabolism, omics, proteome, transcriptome

## Abstract

Leptospirillum ferriphilum plays a major role in acidic, metal-rich environments, where it represents one of the most prevalent iron oxidizers. These milieus include acid rock and mine drainage as well as biomining operations. Despite its perceived importance, no complete genome sequence of the type strain of this model species is available, limiting the possibilities to investigate the strategies and adaptations that Leptospirillum ferriphilum DSM 14647^T^ (here referred to as Leptospirillum ferriphilum^T^) applies to survive and compete in its niche. This study presents a complete, circular genome of Leptospirillum ferriphilum^T^ obtained by PacBio single-molecule real-time (SMRT) long-read sequencing for use as a high-quality reference. Analysis of the functionally annotated genome, mRNA transcripts, and protein concentrations revealed a previously undiscovered nitrogenase cluster for atmospheric nitrogen fixation and elucidated metabolic systems taking part in energy conservation, carbon fixation, pH homeostasis, heavy metal tolerance, the oxidative stress response, chemotaxis and motility, quorum sensing, and biofilm formation. Additionally, mRNA transcript counts and protein concentrations were compared between cells grown in continuous culture using ferrous iron as the substrate and those grown in bioleaching cultures containing chalcopyrite (CuFeS_2_). Adaptations of Leptospirillum ferriphilum^T^ to growth on chalcopyrite included the possibly enhanced production of reducing power, reduced carbon dioxide fixation, as well as elevated levels of RNA transcripts and proteins involved in heavy metal resistance, with special emphasis on copper efflux systems. Finally, the expression and translation of genes responsible for chemotaxis and motility were enhanced.

**IMPORTANCE** Leptospirillum ferriphilum is one of the most important iron oxidizers in the context of acidic and metal-rich environments during moderately thermophilic biomining. A high-quality circular genome of Leptospirillum ferriphilum^T^ coupled with functional omics data provides new insights into its metabolic properties, such as the novel identification of genes for atmospheric nitrogen fixation, and represents an essential step for further accurate proteomic and transcriptomic investigation of this acidophile model species in the future. Additionally, light is shed on adaptation strategies of Leptospirillum ferriphilum^T^ for growth on the copper mineral chalcopyrite. These data can be applied to deepen our understanding and optimization of bioleaching and biooxidation, techniques that present sustainable and environmentally friendly alternatives to many traditional methods for metal extraction.

## INTRODUCTION

The Leptospirillum genus comprises four described species of Gram-negative, chemolithoautotrophic, and acidophilic bacteria: Leptospirillum ferrooxidans (group I) (1), Leptospirillum rubarum (group II) (2), and Leptospirillum ferriphilum and “Leptospirillum ferrodiazotrophum” (group III) (2, 3). In addition, community genomics has identified a further candidate species, “Leptospirillum sp. group IV UBA BS” (2, 4). The original description of the L. ferriphilum type strain gives a temperature optimum of 30°C to 37°C, although many isolated strains are defined as being moderately thermophilic (reviewed in reference 5). Leptospirillum ferriphilum DSM 14647^T^ (here referred to as Leptospirillum ferriphilum^T^) is an obligate aerobe that is capable of gaining energy only via ferrous iron (Fe^2+^) oxidation (3). Finally, it has a pH optimum of 1.4 to 1.8, which requires the cells to maintain an internal, cytoplasmic pH close to neutral in the face of an ∼10^4^-fold proton gradient across the cytoplasmic membrane. As a result, acidophiles have several pH homeostasis mechanisms, including primary (1°) and secondary (2°) proton pumps, an inside positive membrane potential that hinders the influx of protons, proton-consuming reactions, and a cytoplasmic buffering capacity (reviewed in reference 6). Although several Leptospirillum spp. have been identified, current knowledge of how they obtain energy and nutrients for growth is limited. In particular, mechanisms for nitrogen fixation have been under debate. Additionally, the understanding of how members of the leptospirilli survive at acidic pH lags behind that of other acidophiles, such as those from the *Acidithiobacillus* genus (reviewed in references 5 and 7–9).

Leptospirillum spp. are often identified in sulfide mineral-containing environments, where they catalyze the cleavage of the metal sulfide bond by oxidizing ferrous iron (Fe^2+^) back to ferric iron (Fe^3+^) (10). The result of metal sulfide oxidation is an acidic solution typically containing high metal concentrations (reviewed in references 11 and 12). This requires acidophiles to have multiple chemical and biological metal resistance strategies, such as efflux pumps, metal sequestration methods, and the ability to reduce metal uptake via the inside positive membrane potential (reviewed in references 12 and 13). A second consequence of high iron concentrations is the need to mitigate oxidative stress (14), as acidophiles generate intracellular reactive oxygen species (ROS) as well as being exposed to extracellular ROS sources generated by reactions between water or molecular oxygen, dissolved metal ions, and/or surface-bound metal ions on metal sulfides (15, 16). As mentioned above, knowledge of how the leptospirilli survive high metal concentrations and ROS is limited.

The ability to catalyze mineral dissolution has been exploited in the industrial process of “biomining” (reviewed in reference 17), where L. ferriphilum dominates biooxidation tanks for the recovery of gold (3) and has been identified in bioleaching heaps for the recovery of copper from chalcopyrite (CuFeS_2_) (e.g., see reference 18). However, efficient chalcopyrite dissolution in low-cost bioheaps is challenging under mesophilic and moderately thermophilic conditions (19). A critical stage in biomining is cell attachment and biofilm formation on the ore surface (11). Consequently, understanding the genetic basis for cell attachment on metal sulfides may help in the design of strategies to stimulate bioleaching rates, speed up the initiation of bioleaching operations, and improve the persistence of active cells in heap bioleaching operations.

The identification of the genes responsible for biological processes in acidophiles has been hindered until the very recent development of gene knockout systems (e.g., see reference 20), and these methods are still lacking for the leptospirilli. A method to circumvent this limitation is the identification of gene homologs in genome sequences and “metagenome-assembled genomes” (MAGs) that have been used to construct models of individual acidophile strains (reviewed in reference 21) through to community interactions (22). Although several genomes and MAGs from L. ferriphilum strains have been reported (Table 1), the fact that only a draft genome of the L. ferriphilum type strain is available has hindered efforts to elucidate its metabolic properties and evolutionary relationships with the other leptospirilli.

**TABLE 1:** Overview of previously available L. ferriphilum genomes

Strain	Reference or source	NCBI RefSeq accession no.	State of the genome	No. of genes	Genome size (Mbp)	Coding density (%)
L. ferriphilum^T^	24	NZ_JPGK00000000.1	Draft	2,366	2.41	93.1
Sp-Cl	89	NZ_LGSH00000000.1	Draft	2,419	2.48	91.7
YSK	86	NZ_CP007243.1	Complete	2,273	2.33	90.1
ML-04	90	NC_018649.1	Complete	2,475	2.41	90.3
DX	91	NZ_MPOJ00000000.1	Draft	2,324	2.36	85.8
ZJ	91	NZ_MPOK00000000.1	Draft	2,312	2.34	96.4

The present study provides the complete, closed genome of L. ferriphilum^T^ that allows metabolic insights and reveals evolutionary relationships to the leptospirilli and other acidophiles. In addition, we have used RNA transcript sequencing and proteomics to identify the genes used for growth on Fe^2+^ and during biomining of chalcopyrite.

## RESULTS AND DISCUSSION

### General genome data.

**FIG 1:**
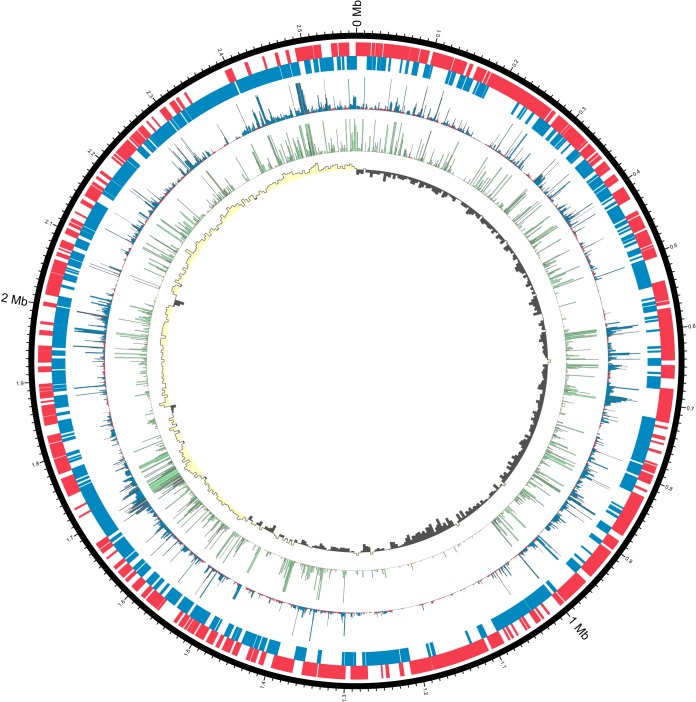
Circular representation (87) of the genome sequence of L. ferriphilum^T^ (configuration in parts based on data in reference 88). From the outside, the bands represent (i) the genome sequence; (ii) protein-encoding sequences on the positive strand (red); (iii) CDSs on the negative strand (blue); (iv) mean transcript expression (TPM), with a maximum of 2,000 TPM (blue indicates TPM values above the median, and red indicates values below the median); (v) mean scaled protein LFQ intensity, with a maximum of 2,000 (green indicates intensity above the median); and (vi) GC-Skew {as calculated by the equation [(C − G)/(C + G)] × 100} in windows of 5,000 nucleotides (yellow indicates values above zero, and gray indicates values below zero).

**FIG 2:**
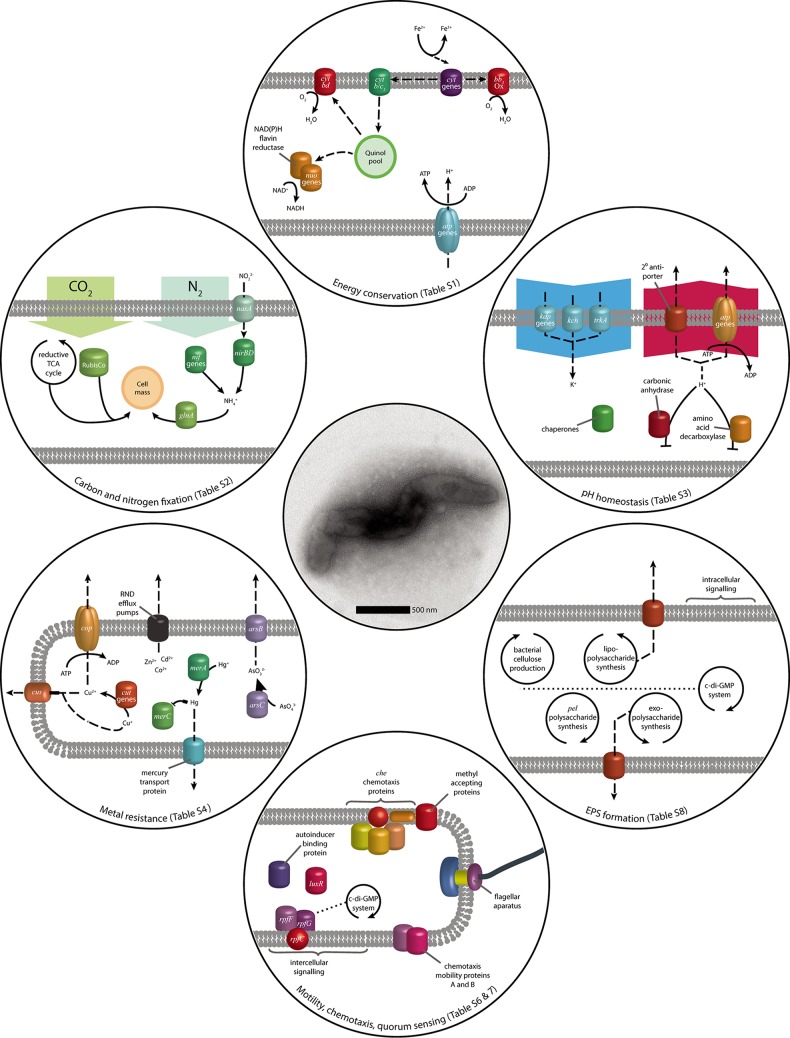
Model of the genomic potential observed in the L. ferriphilum^T^ genome, focusing on functions relevant in acidic environments and its application in biomining (see Tables S1 to S8 in the supplemental material). Solid arrows represent metabolic reactions, while dashed arrows indicate transport, the relocation of electrons or reaction products, and general regulative and metabolic interactions.

**FIG 3:**
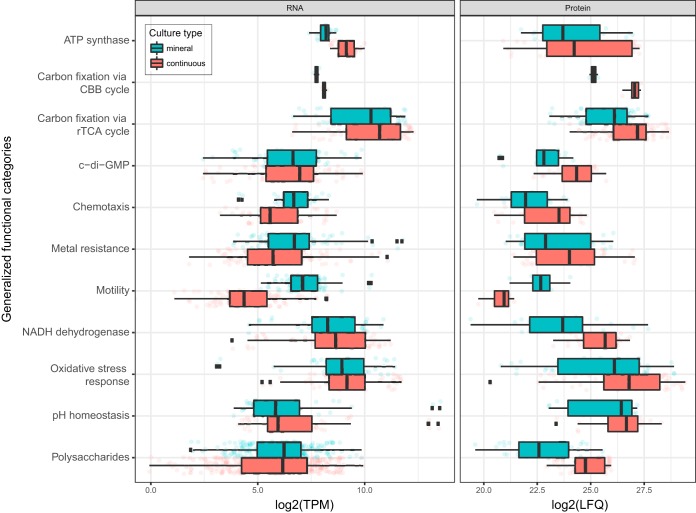
Overview of gene expression values for RNA-seq (TPM) (left) and proteomics (LFQ) (right) with samples grouped according to culture type. The data represent averages of expression values for genes assigned to selected functional categories (based on data in Data Sets S1 to S3 in the supplemental material), with some categories merged to aid comprehension: nitrogen metabolism (ammonia and glutamate conversion to glutamine, nitrate/nitrite regulation, nitrite uptake and assimilation to ammonia, and nitrogenase genes), metal resistance (resistance to arsenic, cadmium/cobalt/zinc, copper, copper/silver, and mercury plus general metal tolerance), polysaccharides (cellulose production, extracellular polysaccharide production and export, and lipopolysaccharide synthesis), c-di-GMP (c-di-GMP effector proteins, with the EAL domain, proteins with the GGDEF domain, and proteins with both the EAL and GGDEF domains), and pH homeostasis (proton-consuming reactions, proton transporters, and role of potassium in internal positive membrane potential). Note that the average translation values depicted are based on various proteins identified in the corresponding samples. Therefore, assumptions about the up- and downregulation of functional categories of proteins have to be made with caution and only in combination with data from differential translation analysis (Fig. 4 and Data Sets S2 and S3). Abbreviation: CBB, Calvin-Benson-Bassham.

The sequencing and assembly of L. ferriphilum^T^ DNA gave two polished contiguous sequences (contigs) (Table 2; see also Report S1 in the supplemental material). Contig 1 was 2,569,357 bases with a depth of coverage of 574-fold, while contig 2 was 41,141 bases with a depth of coverage of 33-fold. Contig 2 was predicted to be a putative phage with VIRSorter (23), and a region on contig 1 with high similarity to contig 2 putatively represents a prophage. Although further analysis is required to determine its origin, contig 2 was excluded due to its low-depth coverage and absence of typical plasmid genes. Circular contig 1 represents the closed chromosome sequence of L. ferriphilum^T^ (Fig. 1). A comparison with the previously available draft genome (24) revealed an additional 163,475 bp, closing gaps in the previous draft (Fig. S1). The most prominent gap with around 100,000 bp most likely had not been previously captured due to the presence of a clustered regularly interspaced short palindromic repeat (CRISPR) stretch (Report S2). Further functionalities in the additional sequences were identified, such as a cluster of *nif* genes. Additional functional capabilities encoded in the L. ferriphilum^T^ genome (Fig. 2) are detailed below, while expressed functions in Fe^2+^-containing medium versus chalcopyrite bioleaching cultures were assessed by transcriptomic and proteomic analyses (Table 3 and Fig. 3). The resulting values are given as transcripts per million base pairs (TPM) for RNA and label-free quantification (LFQ) intensities (25) for proteins, respectively.

**TABLE 2:** General L. ferriphilum^T^ genome statistics

Attribute	Value	% of total
Genome size (bp)	2,569,357	100.00
DNA coding region (bp)	2,331,855	90.76
DNA G+C content (bp)	1,392,384	54.19
Total no. of genes	2,541	100
No. of protein-encoding genes	2,486	97.84
No. of RNA genes (rRNA/tRNA/tmRNA)	6/48/1	0.24/1.93/0.04
No. of CDSs with functional prediction	1,846	74.25
No. of CDSs with assigned COG category	1,969	79.20
No. of CRISPR repeats	1	

**TABLE 3:** Overview of samples and corresponding transcriptomics and proteomics data^*a*^

Sample	Culture type(s)	Total no. of RNA-seq read counts	Median no. of RNA-seq counts	No. of proteins identified	No. of proteins with LFQ of >0	Median LFQ
LNU-LXX9-Si00-CnA-P-B1	Continuous	1,034,434	181	NA	NA	NA
LNU-LXX9-Si00-CnA-P-B2	Continuous	NA	NA	1,698	1,241	160,595,000
LNU-LXX9-Si00-CnA-P-B3	Continuous	NA	NA	1,698	1,509	233,755,000
LNU-LXX9-Si00-CnA-P-B5	Continuous	NA	NA	1,698	1,409	221,875,000
LNU-LXX9-Si00-CnA-P-B6	Continuous	1,284,834	219	1,698	1,412	212,595,000
LNU-LXX9-Si00-CnA-P-B7	Continuous	1,477,391	256	1,698	1,465	217,165,000
LNU-LXX9-Si00-14B-P	Batch, mineral	10,967,703	1,937	763	432	3,135,800
LNU-LXX9-Si00-14C-P	Batch, mineral	12,842,605	2,099	763	513	3,645,200
LNU-LXX9-Si00-14D-P	Batch, mineral	NA	NA	763	609	3,722,500

a NA, not applicable.

### Comparison with other Leptospirillum ferriphilum genomes.

The genomes of six L. ferriphilum strains are available (Table 1), two of which are considered complete. All six genomes show a high degree of identity, with 1,769 orthologous gene clusters conserved in all 6 strains (see Fig. S2 in the supplemental material). The type strain exhibited the largest number of unique gene clusters, which is reflected in the outgrouping of the two type strain sequences in the phylogenetic tree (Fig. S2A). Many of the genes that are distinct for a particular strain seem to be related to insertions or deletions of mobile elements. A cluster of *nif* genes is harbored in the newly sequenced type strain genome and in strains Sp-Cl, YSK, and ZJ. This gene cluster is not present in strains ML-04 and DX and the previous draft of the type strain. The longer stretch (1.0 and 1.3 Mbp) surrounding the *nif* cluster is unique to the type strain, with large parts being present only in the newly sequenced contig (data not shown).

### Energy conservation.

As established above, the energy needs of L. ferriphilum are met exclusively by the oxidation of Fe^2+^ (Fig. 2; see also Table S1 in the supplemental material). Analogous to the iron oxidation system reported previously for L. ferriphilum ML-04, electrons from L. ferriphilum^T^ Fe^2+^ oxidation are transferred to electron carriers (26), which were present on the genome in the form of cytochrome *c*, cytochrome *c*_551/552_, cytochrome *c*_553_, and cytochrome *c*_544_. Thereafter, cytochrome *cbb*_3_ oxidase can be used to directly reduce oxygen as a terminal electron acceptor (27). Alternatively, electrons can be used in reverse electron transport from cytochrome *c* to the quinone pool by the cytochrome *b*/*c*_1_ complex. The resulting quinols can then be used to generate reducing power in the form of NAD(P)H via the NADH-quinone oxidoreductase (*nuoABCDEFHIJKLMN*) (Table S1) or the NAD(P)H-flavin reductase. Although their functionality is unknown, there are also three copies of subunit 5 of NAD(P)H-quinone oxidoreductase (*ndhF*), with which quinols could be used to produce NAD(P)H (28). Finally, electrons from the quinol pool can be transferred to oxygen by using the cytochrome *bd* complex (28), which was also described for ML-04. Proton motive force generated by iron oxidation can be used for ATP generation by an F_o_F_1_-type ATP synthase (*atpABCDEFGH*) (Table S1). RNA transcript counts of the genes involved in energy conservation indicated a preference for cytochrome *c*_551/552_ (639 ± 26 TPM). However, this difference was not observed for the protein concentration. While several genes of all cytochrome groups were only marginally transcribed and translated, no clear trend in the usage of cytochromes as initial electron carriers was apparent (Data Set S1). Further electron transport was likely carried out via *cbb*_3_ cytochromes to oxygen to create a membrane potential for the production of ATP. Although proteins of the competing reverse electron transport chain were expressed, with few exceptions, the pathway utilizing cytochrome *cbb*_3_ had higher transcript counts and protein concentrations than did the pathway utilizing the cytochrome *b*/c_1_ complex and the following quinone pool oxidoreductases (Data Set S1).

### Carbon dioxide fixation.

A single copy of the large-chain subunit of ribulose bisphosphate carboxylase (RubisCO) was encoded on the L. ferriphilum^T^ genome as well as on the genomes of other L. ferriphilum strains. However, all L. ferriphilum strains are suggested to fix carbon via the reductive tricarboxylic acid (TCA) cycle (29), for which all necessary genes were present on the genome (see Table S2 in the supplemental material). This was largely confirmed by transcript and proteome data, as gene products of the reductive TCA cycle were expressed and translated to a high extent (Data Set S1). Although RubisCO (276 ± 14 TPM; LFQ, 27,738 ± 258) exhibited low transcript counts, its protein concentration was comparable to the concentrations of proteins constituting the enzymes of the reductive TCA cycle. However, any role of RubisCO in L. ferriphilum^T^ is unknown.

### Nitrogen fixation.

The nitrogen demand of L. ferriphilum^T^ can be fulfilled by the fixation of elemental nitrogen by the nitrogenase complex *nifABDEHKNTUXZ* (30) and accessory protein genes (see Table S2 in the supplemental material). While present in L. ferrooxidans C2-3 (31) and L. ferriphilum strains Sp-Cl and YSK, this gene cluster was not found in the reported L. ferriphilum^T^ draft genome and likewise is lacking in the complete genome sequence of L. ferriphilum ML-04. Regulatory capabilities for the gene cluster are suggested to be fulfilled by a *nif*-specific regulatory protein in L. ferriphilum^T^. Additionally, nitrogen can be taken up as nitrite by the nitrate/nitrite transporter *nasA* and assimilated in the form of ammonia by the nitrite reductase *nirBD* (32), controlled by regulators of the NtrC and LysR families. RNA transcript analysis of nitrogenase subunits revealed negligible counts, and most of the corresponding proteins were also not detected in the proteomic analysis (Data Set S1). As the growth medium in this study was rich in ammonium, which can be taken up by the highly expressed glutamine synthetase, this was not surprising and has been reported for L. ferrooxidans (33). The highest transcript count within the nitrogen fixation clusters was that for *nifU* (1,997 ± 268 TPM; LFQ, 1,228 ± 58), which is essential for the activation of the nitrogenase complex and is localized together with the cysteine desulfurase gene *nifS* (34). NifS showed the highest protein concentration (661 ± 68 TPM; LFQ, 4,267 ± 175) in the nitrogen fixation clusters despite intermediate transcript counts. In combination with the high expression level of *nifU*, this could indicate an onset of nitrogenase formation due to early-stage ammonium starvation, supported by the intermediate expression of several nitrogen assimilation regulation proteins (Data Set S1).

### pH homeostasis mechanisms.

Acidophiles maintain a near-neutral cytoplasmic pH by several methods, including proton efflux via 1° transport pumps in the electron transport chain (35), and this is discussed in “Energy conservation,” above (see Table S3 in the supplemental material). A second method to maintain pH homeostasis is the inside positive membrane potential that repels the influx of protons (reviewed in reference 6). The internal positive membrane potential is suggested to be formed by K^+^ ions, and this is supported by K^+^-deficient medium inducing acid shock in Sulfolobus acidocaldarius (36). The L. ferriphilum^T^ genome has two copies of the *kdpD*-encoded sensor protein along with the *kdpABC*-encoded K^+^-transporting system as well as two voltage-gated potassium channel genes (*kch* and *trkA*). The Kdp system and the TrkA voltage-gated potassium channel, but not the *kch* gene, were identified on the L. ferriphilum ML-04 genome. In addition, the L. ferriphilum^T^ genome has several 2° proton pumps, such as cation/H^+^ antiporters, and similar antiporter systems were also present on the ML-04 genome. Protons can also be consumed in chemical reactions, such as amino acid decarboxylases in both neutrophiles (37) and acidophiles (35). Three amino acid decarboxylases were identified on the L. ferriphilum^T^ genome, while only glutamate and arginine decarboxylases were present on the L. ferriphilum ML-04 genome. A further proton-consuming reaction encoded in the L. ferriphilum^T^ genome is that of carbonic anhydrase, which has been demonstrated to aid in pH homeostasis (38). A fourth method of pH homeostasis is the production of spermidines that, among other functions (e.g., see “Oxidative stress management,” below), reduce membrane permeability to protons (39), and three genes related to spermidine production were present on the L. ferriphilum^T^ genome. Finally, members of the general stress response protect against acid stress (40), including GroEL, ClpBC, Clp protease, and DnaK. Several of these chaperones were previously identified on the L. ferriphilum ML-04 as well as the Leptospirillum sp. group II strain CF-1 (41) genomes. With the exception of those with additional functions, few of the predicted pH homeostasis genes or proteins had high TPM values or protein levels, respectively (Data Set S1). For instance, the *kdpABC* potassium-transporting genes had TPM values of ≤62 ± 13 and LFQ values of ≤6 ± 2, while the general stress proteins DnaK and GroEL had LFQ values of 6,149 ± 153 and 68,468 ± 6,961, respectively. This suggested that the growth pH of 1.4 did not impose a high level of acid stress on L. ferriphilum^T^.

### Metal resistance systems.

L. ferriphilum^T^ is often exposed to high metal concentrations, and the genome contains the *arsRBC* genes coding for the negative regulator, the arsenite efflux pump, and arsenate reductase, respectively. These genes are present in many acidophiles (reviewed in reference 13), such as L. ferriphilum ML-04 and L. ferriphilum strain Fairview (42). Separate Cu^2+^ and Cu^+^ resistance systems were harbored on the L. ferriphilum^T^ genome, including the copper resistance gene *cop*. This gene can be divided into two functional groups: multicopper oxidases and P-type ATPases used to export copper ions (43). The L. ferriphilum^T^ *cop* gene aligned most closely with sequences of species with confirmed ATPase *cop* activity (data not shown), indicating a similar function in L. ferriphilum^T^. In addition, *cut* is present in the genome for Cu^+^ oxidase as well as the *cusABCF* Cu/Ag system. Cus-like metal resistance systems are part of the *Acidithiobacillus ferrivorans* SS3 mobilome that is thought to reflect selective pressure by the presence of heavy metals (44). A total of 13 open reading frames (ORFs) were determined to encode the RND family Cd^2+^/Co^2+^/Zn^2+^ CzcABCD resistance complex and associated proteins that were also present on the L. ferriphilum ML-04 genome (see Table S4 in the supplemental material). Finally, the mercury resistance *merRAC* genes and a mercury transport protein were identified on the L. ferriphilum^T^ and L. ferriphilum ML-04 genomes. In continuous culture, L. ferriphilum^T^ was grown with only trace concentrations of metals required for cellular metabolism, and consequently, their metal resistance systems were not highly expressed (Data Set S1). In several cases, low metal resistance TPM values were not reflected in concurrent protein production, and this may be due to L. ferriphilum^T^ maintaining a readiness to protect cells against heavy metals. For instance, the expression of the *arsR* negative regulator inhibits the expression of the arsenic resistance operon in many species, including the acidophilic archaeon “*Ferroplasma acidarmanus*” Fer1 (45).

### Oxidative stress management.

Oxidative stress management is crucial for all aerobic organisms. ROS are generated (i) intracellularly via molecular oxygen reactions with metal ions (46); (ii) in the extracellular, acidic, and metal-rich environment of aerobic mineral-oxidizing acidophiles (47); and (iii) on surfaces of metal sulfide minerals (15, 16). Several genes associated with oxidative stress management and ROS degradation were identified in the presented genome sequence (although homologs of catalases and superoxide dismutases were not found [see Table S5 in the supplemental material]). Among these genes, several peroxiredoxins were identified, including genes encoding alkyl hydroperoxide reductase subunit C (*ahpC*), peroxiredoxins (*ccmG* and *dsbE*), and a putative iron-dependent peroxidase (*efeB*). Several thioredoxins, a thioredoxin reductase (*trxB*), and glutaredoxins were also identified. Further genes encoding proteins involved in peroxide degradation are those encoding rubrerythrin and the periplasmic cytochrome *c* peroxidase (48). Furthermore, in the context of an *ahpC* gene and the latter gene, a gene homologous to the transcriptional regulator *perR* was found. Genes involved in the repair or degradation of oxidatively damaged proteins were also identified. ROS degradation and oxidative stress management are also complemented by protective mechanisms such as the production of antioxidants, including spermidine, ectoine, and cobalamin (49, 50). The regulation of metal homeostasis is also involved in the oxidative stress response, and the Fe^3+^ uptake regulator (Fur) family transcriptional regulator and peroxide stress response regulator proteins were identified. Genes for these functions are highly expressed in transcriptomes and proteomes of iron-grown cells (Data Set S1). Expression levels of *ahpC* and the genes encoding further peroxiredoxins, thioredoxin, glutaredoxins, cytochrome *c* peroxidase, and rubrerythrin were especially prominent (Data Set S1). Furthermore, it was also suggested that biofilm formation plus diverse mechanisms of extracellular polysaccharide production and secretion are also part of the L. ferriphilum^T^ ROS defense strategy in a manner similar to that of the Acidithiobacillus ferrooxidans type strain (51), which may be especially relevant during growth on metal sulfide minerals such as chalcopyrite and pyrite.

### Chemotaxis and motility.

Among the mineral dissolution-catalyzing bacteria, Leptospirillum spp. colonize metal sulfide surfaces more efficiently than do the *Acidithiobacilli* (52, 53), and they often comprise a substantial fraction of the community in acid mine drainage streamer biofilms (54, 55). Attached cells on solid metal sulfides are considered to enhance the oxidation of the mineral that serves as an energy source and substratum for mineral-oxidizing bacteria (56, 57). The regulation of biofilm formation involves chemotaxis and motility (see Table S6 in the supplemental material), intracellular signaling via c-di-GMP and intercellular quorum sensing (Table S7), and the production of extracellular polymeric substances (EPSs) (Table S8).

All genes involved in the assembly of a functional flagellar apparatus and its controlling chemotaxis system were identified in the presented genome sequence (Table S6). The L. ferriphilum^T^ chemotaxis system is composed of seven methyl-accepting chemotaxis proteins (MCPs) involved in sensing environmental signals. These genes are scattered across the chromosome, except for LFTS_00227, which was found in the context of the chemotaxis gene cluster. Of the MCPs, only LFTS_01731 was significantly expressed at both the RNA and protein levels in iron-grown chemostat cells. ORFs encoding the flagellar motor switch proteins FliN and FliM were found in different regions on the chromosome. Except for the *fliM* gene, all genes relevant for a functional flagellar motility system were found in two large gene clusters (Table S6). All these genes were found at very low expression levels by using RNA transcript analysis, and either the corresponding protein levels were low or proteins were not detected (Data Set S1), indicating that motility and chemotaxis are of no relevance in a well-mixed, homogenous environment such as a chemostat reactor.

### Quorum sensing and c-di-GMP.

In Gram-negative bacteria, the regulation of genes encoding proteins for chemotaxis, motility, EPS production, and biofilm formation is often controlled by intracellular levels of the messenger molecule c-di-GMP (58). The presented genome sequence provides evidence for complex c-di-GMP metabolism, as is common for many Gram-negative bacteria, including acidophilic mineral-oxidizing *Acidithiobacillus* spp. (59, 60). The L. ferriphilum^T^ genome contains 10 genes annotated as encoding putative diguanylate cyclases, 13 genes encoding both diguanylate cyclase- and c-di-GMP phosphodiesterase-specific GGDEF and EAL protein domains, and two c-di-GMP-specific phosphodiesterases (see Table S7 in the supplemental material). Furthermore, four genes encoding HD/HDc domain-containing proteins and three genes encoding PilZ domain-containing c-di-GMP effector proteins were found. The latter genes were found in the context of genes annotated as being related to functions such as cellulose and extracellular polysaccharide biosynthesis and export. This suggests that c-di-GMP metabolism in L. ferriphilum^T^ also has an important function in the regulation of EPS production and biofilm formation. Several of these genes were expressed at the RNA and protein levels, including a c-di-GMP-specific phosphodiesterase class I-encoding gene, bifunctional diguanylate cyclase/c-di-GMP-specific phosphodiesterase-encoding genes, diguanylate cyclases, and a PilZ domain-containing protein.

Interestingly, the L. ferriphilum^T^ genome contains a gene cluster harboring an *rpf* diffusible signal factor quorum sensing system, which is composed of the diffusible signal factor synthase-encoding gene *rpfF*, two genes encoding *rpfC* homologs annotated as genes encoding the Hpt domain-containing protein and signal transduction kinase, and the respective two-component system response regulator-encoding gene *rpfG*. In addition, further genes related to quorum sensing signaling were identified, such as three *luxR* family transcriptional regulator protein-encoding genes and another autoinducer binding domain-containing gene. The genes encoding the *rpf* quorum sensing system were found to be expressed at enhanced levels, while the orphan LuxR protein-encoding genes were found at very low RNA transcript or protein levels (Data Set S1).

### Biofilm formation.

A total of 103 genes were annotated with functions related to sugar processing, polysaccharide biosynthesis, and export and may be involved in the synthesis of polysaccharides as a constituent of EPSs (see Table S8 in the supplemental material). Among these genes, 47 represent or were in the context of genes primarily associated with lipopolysaccharide synthesis. Several of these genes were found to be expressed in iron-grown chemostat cells (Data Set S1). Interestingly, two gene clusters contain bacterial cellulose synthesis genes, and one of them also contains a gene encoding a cellulase family 8 protein, suggesting that aside from a structural component of EPSs and/or cell walls, cellulose may be used as an intracellular sugar storage compound in L. ferriphilum^T^. Furthermore, a gene cluster highly similar to the Pseudomonas aeruginosa *pel* operon was found. This cluster is responsible for Pel polysaccharide production as part of its EPS constituents. Further genes associated with extracellular polysaccharide export and biosynthesis were found in a large cluster. Among these ORFs, poly-β-1,6-*N*-acetyl-d-glucosamine (PGA) synthesis and export protein-encoding genes were found directly next to a *wza* polysaccharide outer membrane export protein-encoding gene plus further genes with functions associated with polysaccharide assembly and export. In addition, 12 of the ORFs in this cluster were determined to encode glycosyltransferases. However, the majority of these genes were found at very low transcript levels, while the corresponding proteins were not detected in the chemostat (Data Set S1). An exception to the low RNA transcript levels but high protein levels was observed for a UTP-glucose-1-phosphate uridylyltransferase. The *rfbBAC* genes were found in one gene cluster close to an *algK* homolog that is a recently described outer membrane secretin that differs from canonical bacterial capsular polysaccharide secretion systems (61) and a *wzzE* polysaccharide chain length modulation protein. Furthermore, ORFs were determined to encode undecaprenyl (UDP)-galactose-4-epimerases, UDP-galactopyranose mutase, UDP-glucuronate-4-epimerase, and UDP-*N*-acetyl-d-glucosamine dehydrogenase (*wbpA*).

### Biomining lifestyle.

**FIG 4:**
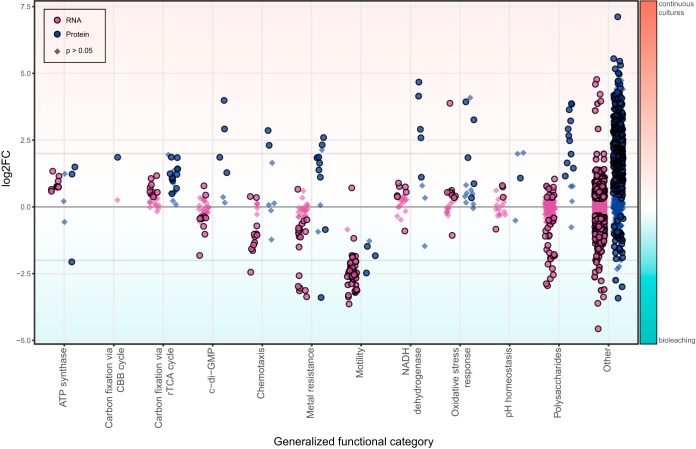
Overview of differential expression (log_2_-fold change) of RNA transcripts and protein concentrations between continuous culture and chalcopyrite-containing bioleaching cultures. Data points represent single transcripts or protein signals, categorized as explained in the legend to Fig. 3. Circular symbols denote statistically significant differences (*P* < 0.05), while diamonds indicate statistically insignificant data.

Bioleaching experiments using pure cultures of L. ferriphilum^T^ achieved a significant dissolution of chalcopyrite (see Fig. S3 in the supplemental material). The isolation of nucleic acids and proteins proved to be challenging, and only two RNA extracts and three protein samples of mineral origins were of sufficient quality for differential expression and translation analyses (Fig. 4 and Data Sets S2 and S3). Owing to the lower sensitivity and dynamic range of the Orbitrap Elite instrument, fewer low-abundance proteins were quantified in the bioleaching samples than in the continuous-culture samples. This manifested as an apparently higher expression level of such gene products in continuous cultures. Therefore, more studies will have to be conducted to confirm the data presented below. To investigate important features and adaptation strategies of L. ferriphilum^T^, RNA transcripts and proteins were grouped based on the functional categories established as described above (Fig. 2 and 3). Comparison of continuous versus mineral culture samples revealed unexpectedly few differences in expression and translation patterns. In part, this is probably related to the controlled nature of the bioleaching experiments, where, e.g., the initial pH was 1.8 and did not decrease below 1.7 (data not shown), such that pH homeostasis systems seemed unaffected by the presence of chalcopyrite. Longer retention times and the presence of sulfur oxidizers would cause the pH to drop significantly (19, 62). Despite the remarkable tolerance of L. ferriphilum^T^ to high proton concentrations (63), this would likely cause additional stress. Similarly, RNA transcript levels and protein concentrations for genes related to nitrogen fixation were found to be stable under the two conditions, conceivably as the culture medium contained large amounts of biologically available ammonium.

Among the differences observed between continuous and bioleaching cultures were decreased transcript counts related to ATP synthesis in the mineral samples along with bidirectional alterations of protein concentrations in ATP synthesis (Fig. 4) and of specific cytochromes and cytochrome oxidases (Data Set S1). This possibly indicated a shift of electron transport away from proton motive force and ATP generation toward the production of reducing power in the form of NAD(P)H (Fig. 2). However, this was not observable in NADH dehydrogenase RNA transcript counts. In contrast, the protein concentration related to NADH production was decreased in the bioleaching experiments (Fig. 4). Additionally, RNA and protein analysis revealed slight reductions in the levels of proteins involved in both above-mentioned carbon fixation pathways when cells were grown on chalcopyrite (Fig. 4). While the exact reasons for this are unknown, it could indicate a reduced demand for organic carbon, possibly caused by overall slow growth along with a reallocation of efforts for cell maintenance under stress conditions in mineral batch cultures compared to active growth in continuous cultures.

Growth on minerals naturally comes with a heightened exposure of cells to heavy metals. Overall, transcript counts derived from metal resistance genes showed significantly increased levels during growth in chalcopyrite bioleaching cultures, in particular a strong enhancement of counts mapping to copper resistance systems (Fig. 4 and Data Set S2). Surprisingly, protein concentrations appeared to be decreased, with two exceptions. In-depth analysis revealed severely elevated amounts of proteins belonging to the *cus* copper efflux system (Fig. 4 and Data Set S3), underlining the strong detrimental effects of copper ions on microbes (64). Similar to the pH homeostasis response, as metal concentrations increase with time in natural or industrial systems, further upregulation of these systems should be expected.

Damage caused by heavy metal ions can often be mitigated by oxidative damage repair systems (65). The majority of these genes were found to exhibit the same or even fewer transcript counts and protein levels in mineral-grown cells (Fig. 4 and Data Set S3). This was surprising, as they have been suggested to combat the heightened oxidative damage caused by ROS produced at the mineral surface (15, 16). An explanation for this behavior could be that the combined effect of high Fe^3+^ concentrations and excessive sparging with air in continuous culture induced more oxidative damage than ROS produced on mineral surfaces.

L. ferriphilum^T^ was previously reported to rapidly attach to mineral surfaces (52), and RNA transcript counts of both chemotaxis and motility systems were revealed to be heavily enhanced during the bioleaching experiments. This was also observed for motility protein concentrations but not chemotaxis protein concentrations (Fig. 4 and Data Set S2). The transcription and translation of c-di-GMP and EPS production remained at the same or lower levels in mineral culture samples (Fig. 4). However, this may be explained by the fact that sampling of mineral-grown cells was conducted on the slowly agitated overlying medium and not the biofilm on the mineral grains, where most of the biofilm regulation and EPS production are expected to occur (56, 66). In contrast, samples taken from the continuous culture were well mixed and likely contained both planktonic and detached biofilm cells.

### Conclusions.

The newly sequenced genome of L. ferriphilum^T^ allows an in-depth and complete characterization of this organism's metabolic potential as well as its expression and translation behaviors in continuous culture and batch bioleaching experiments. PacBio single-molecule real-time (SMRT) long-read sequencing allowed the assembly of a circular chromosome and revealed key features of the adaptation of L. ferriphilum^T^ to acidic, metal-rich environments associated with sulfidic minerals, in the environment as well as in industrial applications. Additionally, RNA transcript sequencing and protein identification elucidated stressing factors during chalcopyrite biomining and shed light on resistance systems deployed by L. ferriphilum^T^. The data provided by this study pose a valuable resource for future experiments investigating the role of L. ferriphilum^T^ in acid mine and rock drainage as well as bioleaching processes.

## MATERIALS AND METHODS

### Batch cell culture and DNA extraction.

The Leptospirillum ferriphilum type strain (ATCC 49881 and DSM 14647) was cultured aerobically at 37°C at pH 1.5 to 1.6 in MAC medium (67) containing 100 mM sterile-filtered (0.2-μm filter) Fe^2+^ as the electron donor. Cells were grown to late log phase before harvesting at 10,000 × *g* for 10 min. DNA for sequencing was isolated by using the Genomic-tip 100/G extraction kit (Qiagen) according to the manufacturer's instructions, with the exception of a customized purification step recommended by the sequencing facility. Briefly, eluted genomic DNA was precipitated by the addition of isopropanol, immediately spooled by using a sterile pipette tip, and transferred to a microcentrifuge tube containing 70% (vol/vol) ethanol for 2 min. Spooled DNA was then air dried, finally resuspended in 200 μl 0.1× Tris-EDTA (TE) buffer (pH 8), and allowed to dissolve for 72 h at room temperature.

### Continuous cultivation, bioleaching experiments, and RNA and protein isolation.

L. ferriphilum^T^ was grown in a substrate-limited, 1-liter-working-volume, continuous-culture vessel at 37°C. The electron donor was provided in the form of MAC medium containing sterile-filtered 100 mM Fe^2+^ (dilution rate [*D*] = 0.3 liters/day). The pH of the medium was adjusted to pH 1.1 by the addition of sulfuric acid that maintained a constant pH of 1.4 within the culture. For the collection of RNA and protein, replicate 100-ml samples were taken from the cultures at least 3 days apart. To minimize RNA degradation, samples were rapidly cooled by mixing with 1 volume of ice-cold sterile MAC medium, and cells were immediately pelleted by centrifugation at 4°C at 12,000 × *g* for 15 min. The cells were then washed in 40 ml fresh, ice-cold MAC medium before being centrifuged again. Finally, pellets were flash-frozen in liquid nitrogen and stored at −80°C for the extraction step.

Additionally, L. ferriphilum^T^ was cultured in four bioleaching flasks containing 100 ml MAC medium (pH 1.8) supplemented with 2% (wt/vol) copper mineral chalcopyrite (CuFeS_2_) as the only energy source. Mixtures for the bioleaching experiments were incubated for 14 days under slow shaking (120 rpm). Seventy-five milliliters of the overlying medium was taken as a sample and processed as described above.

Cell pellets were subjected to biomolecular extractions based on a previously reported protocol (68), skipping the metabolite extraction step. In short, cell pellets were lysed by cryo-milling and bead beating followed by the column-based isolation of biomolecules with the Qiagen Allprep kit. Quality control was performed with SDS-PAGE (protein) and measurements on an Agilent bioanalyzer (total RNA).

### DNA sequencing and genomic analysis.

The obtained genomic DNA was sent to the Science for Life Laboratory (Stockholm, Sweden) and sequenced by using two PacBio SMRT cells. Assembly was conducted with HGAP3 at the sequencing facility, including quiver for consensus corrections. The large contig was circularized with Circlator (69) after inspection of dot plots produced with Gepard (70). The −fixstart option was applied to set the *dnaA* gene as the first gene. Prokka v1.12-beta (71) was used for genome annotation, which included Prodigal v2.6.3 (72) for the prediction of protein-encoding sequences. Functional annotation of coding sequences (CDSs) was performed with a custom genus database of related genomes downloaded from the Integrated Microbial Genomes (IMG) system (73): Leptospirillum sp. group IV UBA BS (GOLD identification Ga0053748 [https://gold.jgi.doe.gov/analysis_projects?id=Ga0053748]), “*Leptospirillu*m sp. group II C75” (GOLD identification Ga0039193 [https://gold.jgi.doe.gov/analysis_projects?id=Ga0039193]), L. ferrooxidans C2-3 (NCBI RefSeq accession number AP012342), L. ferriphilum ML-04 (NCBI RefSeq accession number CP002919), L. ferriphilum DSM 14647 (GOLD identification Ga0059175 [https://gold.jgi.doe.gov/analysis_projects?id=Ga0059175]), and L. ferriphilum YSK (NCBI RefSeq accession number CP007243). Protein sequences were searched (blastp) in Prokka against this genus database, and annotations of best-matching hits were transferred with an E value cutoff of 1e−9. Additionally, the standard databases in Prokka were searched. For additional information, in-house hidden Markov model (HMM) databases were searched, including KEGG orthologous groups (KOs), PFAM, TIGRFAM, UniProt-enzymes, and MetaCyc (additional details are available in reference 74). Furthermore, the annotation tool Pannzer (75) was applied. Additional annotations are listed in Data Set S1 in the supplemental material. Functional categories were assigned based on the KO annotation (COG, KEGG class). Additionally, genes were grouped into functional categories by manual assignment.

### RNA sequencing and transcript analysis.

RNA samples were adjusted for equimolar concentrations and sent to the Science for Life Laboratory (Stockholm, Sweden). Library preparation was performed with the Illumina TruSeq Stranded total RNA kit. Paired-end sequencing was performed on one HiSeq2500 lane for a total of five L. ferriphilum^T^ samples, three from continuous cultures and two successful extracts from batch cultures with chalcopyrite. Batch culture samples were depleted of rRNA with the bacterial Ribo-Zero rRNA removal kit (Illumina) prior to library preparation.

A custom pipeline was written in snakemake (76) for processing and analysis of the transcriptome sequencing (RNA-seq) data (the source code is available at https://git-r3lab.uni.lu/malte.herold/LF_omics_analysis). Raw reads for RNA sequencing were preprocessed with Trimmomatic v0.36 (77) with the file TruSeq3-PE.fa for adapters. Preprocessed reads were mapped onto a concatenation of reference genomes of three acidophiles, including the newly assembled chromosome of L. ferriphilum^T^, with bowtie2 v2.3.2 with default settings. Read mappings to CDSs were counted with the software featureCounts from subread package v1.5.2 (78), and the −s 2 option was used to include only reads on the correct strand. Raw read counts were normalized to the gene length and the sum of total counted reads. Normalized counts were represented as transcripts per million base pairs (TPM). Raw counts for the CDS features of continuous and batch culture samples were subjected to differential analysis with DeSeq2 v1.16.1 (79).

### Proteomics and protein identification.

Five separate protein extracts from a continuous culture and three batch cultures were precipitated in acetone, dried, and then dissolved in 20 μl of 6 M urea–2 M thiourea by vortexing. The reduction of cysteine was done by incubation with 1 μl 1 M dithiothreitol for 30 min at room temperature. Cysteines were alkylated with 1 μl 550 mM iodoacetamide for 20 min in the dark. Proteins were then digested with lysyl endopeptidase (Wako) at a 1:100 protease/protein ratio at room temperature for 3 h. Upon the dilution of urea to 2 M with 50 mM ammonium bicarbonate, further digestion occurred with trypsin (sequencing grade; Promega) at a protease/protein ratio of 1:100 at room temperature for 12 h. Peptides were extracted from the gel pieces with acetonitrile, loaded onto stop-and-go extraction (STAGE) tips for storage, and eluted from the tips shortly before mass spectrometry (MS) analysis (80).

Mass spectrometry for continuous-culture samples was carried out by using an EASY-nLC 1000 liquid chromatography (LC) system (Thermo Scientific) and a Q-Exactive HF mass spectrometer (Thermo Scientific), as described previously (81). Mass spectra were recorded with Xcalibur software 3.1.66.10 (Thermo Scientific). Mass spectrometry for mineral culture samples was carried out by using a nanoACQUITY gradient ultraperformance liquid chromatography (UPLC) pump system (Waters, Milford, MA, USA) coupled to an LTQ Orbitrap Elite mass spectrometer (Thermo Fisher Scientific Inc., Waltham, MA, USA). An UPLC HSS T3 M-class column (1.8 μm, 75 μm by 150 mm; Waters, Milford, MA, USA) and an UPLC Symmetry C_18_ trapping column (5 μm, 180 μm by 20 mm; Waters, Milford, MA, USA) were used for LC in combination with a PicoTip emitter (SilicaTip, 10-μm internal diameter [i.d.]; New Objective, Woburn, MA, USA). For elution of the peptides, a linear gradient with increasing concentrations of buffer B (0.1% formic acid in acetonitrile [ULC/MS grade]; Biosolve, Netherlands) from 1% to 95% within 166.5 min was applied, followed by a linear gradient from 1% acetonitrile within 13.5 min (1% buffer B from 0 to 10 min, 5% buffer B from 10 to 161 min, 40% buffer B from 161 to 161.5 min, 85% buffer B from 161.5 to 166.5 min, 95% buffer B from 166.5 to 167.1 min, and 1% buffer B from 167.1 to 180 min) at a flow rate of 400 nl min^−1^ and a spray voltage of 1.5 to 1.8 kV. The column was reequilibrated with 2% buffer B within 15 min. The analytical column oven was set to 55°C, and the heated desolvation capillary was set to 275°C. The LTQ Orbitrap Elite instrument was operated by using instrument method files of Xcalibur (Rev.2.1.0) in the positive-ion mode. The linear ion trap and Orbitrap instruments were operated in parallel; i.e., during a full MS scan on the Orbitrap instrument in the range of 150 to 2,000 *m/z* at a resolution of 60,000, tandem MS (MS/MS) spectra of the 10 most intense precursors, from the most intense to the least intense, were detected in the ion trap. The relative collision energy for rapid collision-induced dissociation (rCID) was set to 35%. Dynamic exclusion was enabled with a repeat count of 1 and a 45-s exclusion duration window. Singly charged ions and ions of an unknown charge state were rejected for MS/MS. Mass spectra were recorded with Xcalibur software 2.2 SP1.48 (Thermo Scientific).

Proteins under both culture conditions were identified with Andromeda (82) and quantified with the LFQ algorithm (25) embedded in MaxQuant version 1.5.3.175 (81). The FASTA protein database for identification was taken from the output of the functional annotation of the chromosome and contained 2,486 entries. After quantification, intensities from the LFQ normalization were filtered and compared with Perseus (v1.5.8.5) (83), removing rows with fewer than two values under either condition (mineral or continuous). The two conditions were compared with two-sample Welch's *t* test.

### Data availability.

DNA raw sequencing data and the resulting assembly are available under BioProject accession number PRJEB21703 and Assembly accession number GCA_900198525.1. Raw reads for transcriptome sequencing are available under BioProject accession number PRJEB21842. Links to raw data repositories, processed data files, and repositories containing the respective computational workflows are available through the fairdomhub platform (84) in a structured format (see reference 85 [https://doi.org/10.15490/fairdomhub.1.investigation.162.1]).

## Supplementary Material

Supplemental material
