# Supplementary material for: Multi-omics Reveals the Lifestyle of the Acidophilic, Mineral-Oxidizing Model Species Leptospirillum ferriphilumT
Source: Appl Environ Microbiol. 2018 Jan 17;84(3):e02091-17. doi: 10.1128/AEM.02091-17 (PMC5772234; doi:10.1128/AEM.02091-17)
Supplement: Supplemental material [file supp_84_3_e02091-17__index.html]

Supplemental material 

# Multi-omics Reveals the Lifestyle of the Acidophilic, Mineral-Oxidizing Model Species Leptospirillum ferriphilumT

## Supplemental material

- Supplemental file 1 -

  Genes attributed to energy conservation identified in the *Leptospirillium ferriphilum* DSM 14647T genome (Table S1); carbon dioxide and nitrogen fixation genes identified in the *Leptospirillium ferriphilum* DSM 14647T genome (Table S2); genes attributed to adaptation to growth at low pH identified in the *Leptospirillium ferriphilum* DSM 14647T genome (Table S3); metal resistance genes (Table S4), oxidative stress response genes (Table S5), chemotaxis and motility genes (Table S6), quorum sensing and c-di-GMP genes (Table S7), and biofilm formation genes (Table S8) identified in the *Leptospirillium ferriphilum* DSM 14647T genome; Circoletto plot showing a comparison between the new assembly contig1 and the 18 contigs of the draft genome (Fig. S1); phylogenetic placement and relationship of *L. ferriphilum* strains (Fig. S2); redox potential (A) and release of copper and iron (B) from chalcopyrite concentrate during bioleaching by *L. ferriphilum*T compared to a sterile control (Fig. S3); details of the PacBio sequencing run (Report S1); summary of CRISPR and phage association predictions for contig1 and contig2 (Report S2).

  PDF, 1.7M
- Supplemental file 2 -

  Quantification of RNA transcripts and protein concentration identified in *Leptospirillium ferriphilum* DSM 14647T during growth in continuous culture and in bioleaching cultures using chalcopyrite as substrate (Data Set S1).

  XLSX, 1.1M
- Supplemental file 3 -

  DeSeq2 comparison of RNA read counts identified in *Leptospirillium ferriphilum* DSM 14647T grown in continuous culture versus bioleaching cultures using chalcopyrite as substrate (Data Set S2).

  XLSX, 340K
- Supplemental file 4 -

  Comparison of protein intensities (LFQ) identified in *Leptospirillium ferriphilum* DSM 14647T grown in continuous culture versus bioleaching cultures using chalcopyrite as substrate (Data Set S3).

  XLSX, 159K
