# Supplementary material for: Multi-omics Reveals the Lifestyle of the Acidophilic, Mineral-Oxidizing Model Species Leptospirillum ferriphilumT
Source: Appl Environ Microbiol. 2018 Jan 17;84(3):e02091-17. doi: 10.1128/AEM.02091-17 (PMC5772234; doi:10.1128/AEM.02091-17)
Supplement: Supplemental material [file AEM.02091-17_zam003188291s1.pdf]

Multi-omics reveal the lifestyle of acidophile, mineral-oxidizing model species *Leptospirillum ferriphilum*^{T}

SUPPLEMENTAL MATERIAL

Stephan Christel[a], Malte Herold[b], Sören Bellenberg[c], Mohamed El Hajjami[d], Antoine Buetti-Dinh[e,f], Igor Pivkin[e,f], Wolfgang Sand[c,g,h] , Paul Wilmes[b], Ansgar Poetsch[d,i], and Mark Dopson[a,#]

Centre for Ecology and Evolution in Microbial Model Systems, Linnaeus University, Kalmar, Sweden[a]; Luxembourg Centre for Systems Biomedicine, University of Luxembourg, Belvaux, Luxembourg[b]; Aquatic Biotechnology, University Duisburg-Essen, Essen, Germany[c]; Plant Biochemistry, Ruhr University Bochum, Germany[d]; Institute of Computational Science, Faculty of Informatics, Università della Svizzera Italiana, Lugano, Switzerland[e]; Swiss Institute of Bioinformatics, Lausanne, Switzerland[f]; College of Environmental Science and Engineering, Donghua University, Shanghai, PR China[g]; Mining Academy and Technical University Freiberg, Freiberg, Germany[h]; School of Biomedical and Healthcare Sciences, Plymouth University, UK[i]

#Address correspondence to Mark Dopson, mark.dopson@lnu.se

S.C. and M.H. contributed equally to this work

TABLE S1 Genes attributed to energy conservation identified in the *Leptospirillium ferriphilum*^{T} DSM 14647 genome.

TABLE S2 Carbon dioxide and nitrogen fixation genes identified in the *Leptospirillium ferriphilum*^{T} DSM 14647 genome. Genes for other carbon dioxide fixation pathways (i.e. Calvin- Benson-Bassham (CBB) cycle, reductive acetyl-coenzyme A pathway, and 3-hydroxpropionate cycle) were either not identified or key genes required for the pathway were missing.

TABLE S3 Genes attributed to adaptation to growth at low pH identified in the *Leptospirillium ferriphilum*^{T} DSM 14647 genome. Primary proton pumps that export protons are listed in Table S1 while general stress response genes have only been included where a link to pH homeostasis has been reported.

TABLE S4 Metal resistance genes identified in the *Leptospirillium ferriphilum*^{T} DSM 14647 genome.

TABLE S5 Oxidative stress response genes identified in the *Leptospirillium ferriphilum*^{T} DSM 14647 genome.

TABLE S6 Chemotaxis and motility genes identified in the *Leptospirillium ferriphilum*^{T} DSM 14647 genome.

TABLE S7 Quorum sensing and c-di-GMP genes identified in the *Leptospirillium ferriphilum*^{T} DSM 14647 genome.

TABLE S8 Biofilm formation genes identified in the *Leptospirillium ferriphilum*^{T} DSM 14647 genome.

FIG S1 Circoletto plot (Darzentas et al, 2010,. http://tools.bat.infspire.org/circoletto/ 10/01/2015) showing a comparison between the new assembly contig1 (white) and the 18 contigs of the draft genome (24). Default settings were used and colored ribbons indicate relative alignment scores which in this case primarily reflect sequence length.

FIG S2 Phylogenetic placement and relationship of L. ferriphilum strains. Shown are an evolutionary tree of the available strains(A), and a Venn diagram of orthologous gene clusters (B). The phylogenetic tree was constructed with CVTree3 (3) (http://tlife.fudan.edu.cn/archaea/cvtree/cvtree3/ 30/10/2017) using amino acid sequences

with a K-tuple length of 6. Venn diagram was created with OrthoVenn (4) (http://www.bioinfogenome.net/OrthoVenn/index.php 30/10/2017) using protein sequences downloaded from NCBI as input. More detailed analysis as well as functional categories assigned to shared genes can be found under url http://www.bioinfogenome.net/OrthoVenn/result.php?ID=d0faeba113424e41958bda82bce23f37.

FIG S3 Redox potential (A) and release of copper and iron (B) from chalcopyrite concentrate during bioleaching by *L. ferriphilum*^{T} compared to a sterile control.

REPORT S1 Details of the PacBio sequencing run.

REPORT S2 Summary of CRISPR and phage association predictions for contig1 and contig2.

Supplemental references

TABLE S1 Genes attributed to energy conservation identified in the *Leptospirillium ferriphilum*^{T} DSM 14647 genome.

**Locus Tag**	**Gene**	**Predicted Function**
**NADH dehydrogenase**		
LFTS_00278		NAD(P)H-flavin reductase
LFTS_01220<br>LFTS_01821<br>LFTS_01828	*ndhF*	NAD(P)H-quinone oxidoreductase subunit 5
LFTS_00687	*nuoA*	NADH-quinone oxidoreductase subunit A
LFTS_00688	*nuoB*	NADH-quinone oxidoreductase subunit B
LFTS_00689	*nuoC*	NADH-quinone oxidoreductase subunit C
LFTS_00690	*nuoD*	NADH-quinone oxidoreductase subunit D
LFTS_00691	*nuoE*	NADH-quinone oxidoreductase subunit E
LFTS_01451<br>LFTS_01487<br>LFTS_02078	*nuoF*	NADH-quinone oxidoreductase subunit F
LFTS_00694	*nuoH*	NADH-quinone oxidoreductase subunit H
LFTS_00695	*nuoI*	NADH-quinone oxidoreductase subunit I
LFTS_00696	*nuoJ*	NADH-quinone oxidoreductase subunit J
LFTS_00697	*nuoK*	NADH-quinone oxidoreductase subunit K
LFTS_00698<br>LFTS_02316	*nuoL*	NADH-quinone oxidoreductase subunit L
LFTS_00699<br>LFTS_02315	*nuoM*	NADH-quinone oxidoreductase subunit M
LFTS_00700	*nuoN*	NADH-quinone oxidoreductase subunit N
**Succinate dehydrogenase / fumarate reductase**		
LFTS_00585	*sdhA*	Succinate dehydrogenase / fumarate reductase flavoprotein subunit
**Cytochrome *bd***		
LFTS_00439	*cydA*	Cytochrome *d* ubiquinol oxidase subunit I
LFTS_00440	*cydB*	Cytochrome *d* ubiquinol oxidase subunit II
LFTS_00441	*ybgT*	Cytochrome *bd*-I terminal oxidase
Cytochrome b/c_1		
LFTS_00821<br>LFTS_01943	*qcrA*	Menaquinol cytochrome b_6 complex iron-sulfur subunit 1
LFTS_00822	*qcrB*	Menaquinol-cytochrome *c* reductase cytochrome *b* subunit
**Cytochrome *c***		
LFTS_00766		Cytochrome *c* mono- and diheme variants
LFTS_02381	*ccsB*	Cytochrome *c* biogenesis protein
LFTS_02382	*resB*	Cytochrome *c* biogenesis protein
LFTS_01388		Cytochrome $c_{551/552}$
LFTS_00732		Cytochrome c_{553}

LFTS_00740		
LFTS_00940		Cytochrome c_{544}
LFTS_00730<br>LFTS_00731<br>LFTS_00741		Cytochrome *c*
LFTS_02378	*ccdA*	Cytochrome *c* biogenesis protein
**Cytochrome cbb_3 oxidase**		
LFTS_01396<br>LFTS_01857<br>LFTS_01964		Cytochrome *c* oxidase cbb_3-type subunit I
LFTS_01531<br>LFTS_01972		Cytochrome *c* oxidase cbb_3-type subunit II
LFTS_01351<br>LFTS_01930<br>LFTS_02094<br>LFTS_02276		Cytochrome *c* oxidase cbb_3-type subunit III
**ATP synthase**		
LFTS_00066	*atpA*	ATP synthase subunit alpha
LFTS_00062	*atpB*	ATP synthase subunit a
LFTS_00069	*atpC*	ATP synthase epsilon chain
LFTS_00068	*atpD*	ATP synthase subunit beta
LFTS_00063	*atpE*	ATP synthase subunit c
LFTS_00064	*atpF*	ATP synthase subunit b
LFTS_00067	*atpG*	ATP synthase gamma chain
LFTS_00065	*atpH*	ATP synthase subunit delta
LFTS_00061		Putative F_0F_1-ATPase subunit Ca^{2+}/Mg^{2+} transporter

TABLE S2 Carbon dioxide and nitrogen fixation genes identified in the *Leptospirillium ferriphilum*[T] DSM 14647 genome. Genes for other carbon dioxide fixation pathways (i.e. Calvin-Bassham-Benson (CBB) cycle, reductive acetyl-coenzyme A pathway, and 3-hydroxpropionate cycle) were either not identified or key genes required for the pathway were missing.

Locus Tag	Gene	Predicted Function
**Reductive TCA cycle carbon dioxide fixation**		
ROKKA_02486		Phosphoenolpyruvate carboxylase
LFTS_00876	*mdh2*	Malate dehydrogenase (NAD)
LFTS_01556	*fumC*	Fumarase class II
LFTS_00585		Succinate dehydrogenase / fumarate reductase flavoprotein subunit
LFTS_00579	*sucD*	Succinyl-CoA synthetase alpha subunit
LFTS_00587		
LFTS_00586	*sucC*	Succinyl-CoA synthetase beta subunit
LFTS_02458	forA2	pyruvate ferredoxin oxidoreductase alpha subunit
LFTS_02457	forB2	pyruvate ferredoxin oxidoreductase beta subunit
LFTS_02451	forG1	pyruvate ferredoxin oxidoreductase gamma subunit
LFTS_02456	forG2	pyruvate ferredoxin oxidoreductase gamma subunit
LFTS_00965		Isocitrate dehydrogenase (NAD^+)
LFTS_00966		Putative isocitrate dehydrogenase (NADP)
LFTS_00581		Aconitase
LFTS_00582		Crityl-CoA lyase
LFTS_00578		Crityl-CoA synthetase large subunit
LFTS_02453	*forA*	Pyruvate ferredoxin oxidoreductase alpha subunit
LFTS_02452	*forB*	Pyruvate ferredoxin oxidoreductase beta subunit
LFTS_02455	*forD*	Pyruvate:ferredoxin oxidoreductase delta subunit
LFTS_02450		Pyruvate ferredoxin oxidoreductase epsilon subunit
LFTS_02143	*ppsA*	Phosphoenolpyruvate synthase
**Calvin-Benson-Bassham cycle carbon dioxide fixation**		
LFTS_02524	*rbcL*	Ribulose-bisphosphate carboxylase large chain
**Nitrate/nitrite regulation**		
LFTS_00042	*ntrX*	NtrC family, nitrogen regulation response regulator
LFTS_01867	*glnG*	NtrC family nitrogen regulation response regulator
LFTS_00534		
LFTS_01618		LysR family transcriptional regulator nitrogen assimilation regulator
LFTS_00311		Nitrogen regulatory protein P-II family protein
LFTS_00704		
LFTS_01184		
LFTS_01185		
LFTS_01200		

LFTS_01203<br>LFTS_02313		
LFTS_02317		Nif-specific regulatory protein
**Nitrite uptake & assimilation to ammonia**		
LFTS_01202	*nasA*	MFS transporter NNP family nitrate/nitrite transporter
LFTS_01201	*nirB*	Nitrite reductase large subunit
LFTS_02431	*nirD*	Nitrite reductase small subunit
**Nitrogenase genes**		
LFTS_01957	*nifA*	Nif-specific regulatory protein
LFTS_01169	*nifB*	FeMo cofactor biosynthesis protein
LFTS_01163	*nifD*	Mo-nitrogenase MoFe protein alpha subunit
LFTS_01165	*nifE*	Nitrogenase molybdenum-cofactor synthesis protein NifE
LFTS_01162	*nifH*	Nitrogenase iron protein
LFTS_01164	*nifK*	Mo-nitrogenase MoFe protein beta subunit
LFTS_01166	*nifN*	Nitrogenase iron-molybdenum cofactor biosynthesis protein NifN
LFTS_00421<br>LFTS_01174		Cysteine desulfurase
LFTS_01179	*nifT*	Nitrogen fixation protein
LFTS_00420<br>LFTS_01178	*nifU*	Scaffold protein for iron-sulfur cluster assembly
LFTS_01167	*nifX*	Nitrogenase, MoFe protein
LFTS_01177	*nifZ*	Cysteine desulfurase
LFTS_01168		Putative nitrogen fixation protein
LFTS_01182		Ferredoxin *nif*-specific
LFTS_01183		Nitrogenase-stabilizing/protective protein
**Ammonia & glutamate conversion to glutamine**		
LFTS_00312	*amt*	Ammonium transporter
LFTS_00310<br>LFTS_00703	*nrgA*	Ammonium transporter
LFTS_01907	*glnA*	Glutamine synthetase

TABLE S3 Genes attributed to adaptation to growth at low pH identified in the *Leptospirillium ferriphilum*^{T} DSM 14647 genome. Primary proton pumps that export protons are listed in Supplemental File 2 while general stress response genes have only been included where a link to pH homeostasis has been reported.

Locus Tag	Gene	Predicted Function
**Role of potassium in the internal positive membrane potential**		
LFTS_00452	*kdpC*	K^+-transporting ATPase C chain
LFTS_00453	*kdpA*	K^+-transporting ATPase A chain
LFTS_00454	*kdpB*	K^+-transporting ATPase B chain
LFTS_00075<br>LFTS_00459	*kdpD*	Sensor protein gene
LFTS_02015	*kch*	Voltage-gated potassium channel
LFTS_02359	*trkA*	Voltage-gated potassium channel
LFTS_00343		Ca^{2+}-Na^+/H^+ antiporter
LFTS_01531	*chaC*	Cation transport protein
LFTS_00392		Na^+/H^+ antiporter CPA family
LFTS_02006<br>LFTS_02015		Monovalent cation:H^+ antiporter CPA1 family
**Proton consuming reactions**		
LFTS_00495		Glutamate decarboxylase
LFTS_00855	*ldcC*	Arginine decarboxylase
LFTS_02102	*panD*	L-aspartate 1-decarboxylase
LFTS_00030<br>LFTS_00296		Carbonic anhydrase
**Spermidine**		
LFTS_00856	*speE*	Spermidine synthase
LFTS_00857	*speH*	S-adenosylmethionine decarboxylase
LFTS_01989		Glutathionylspermidine synthase preATP-grasp
**Chaperones**		
LFTS_00851	*groL*	Chaperonin GroEL
LFTS_00258	*clpB*	ClpB protein
LFTS_00785<br>LFTS_00927	*clpC*	ATP-dependent Clp protease
LFTS_00839		Clp protease
LFTS_00948		Molecular chaperone DnaK

TABLE S4 Metal resistance genes identified in the *Leptospirillium ferriphilum*^{T} DSM 14647 genome.

Locus Tag	Gene	Predicted Function
**Arsenic resistance**		
LFTS_00324	*arsR*	As transcriptional repressor
LFTS_00753	*arsB*	As efflux membrane subunit
LFTS_00754	*arsC*	Arsenate reductase
**Copper resistance**		
LFTS_02050		Copper-exporting P-type ATPase
LFTS_00313		
LFTS_00752	*copZ*	Periplasmic copper-binding
LFTS_00684	*cutA*	Cu(II) binding protein
**Copper/silver resistance**		
LFTS_00919	*cusA*	Cu(I)/Ag(I) efflux system membrane protein
LFTS_00166	*cusB*	Cu(I)/Ag(I) membrane fusion protein
LFTS_02435		
LFTS_00920		
LFTS_01521		
LFTS_02071	*cusC*	Cu(I)/Ag(I) efflux system, outer membrane component
LFTS_00918	*cusF*	Cu(I)/Ag(I) efflux protein
LFTS_02048		
**Cadmium/cobalt/zinc resistance**		
LFTS_00167	*czcA*	Cd/Co/Zn H^{+}-K^{+} efflux
LFTS_01520		
LFTS_01619		
LFTS_02037		
LFTS_02038	*czcB*	RND family efflux transporter MFP subunit
LFTS_00326		
LFTS_01620		
LFTS_02419		
LFTS_02423		
LFTS_02039	*czcC*	Outer membrane protein cobalt-zinc-cadmium efflux system
LFTS_01521		
LFTS_00665	*czcD*	Cation diffusion facilitator family transporter
LFTS_02043		Cobalt-zinc-cadmium efflux system protein
**Mercury resistance**		
LFTS_00560	*merR*	Mercuric resistance operon regulatory protein
LFTS_00559	*merA*	Mercuric reductase
LFTS_01909		
LFTS_01157		
LFTS_01156	*merC*	Resistance protein
LFTS_00558		Mercuric ion transport protein
**Metal tolerance**		
LFTS_00076		Heavy-metal resistance protein

LFTS_00755		
LFTS_01059		
LFTS_02594		

TABLE S5 Oxidative stress response genes identified in the *Leptospirillium ferriphilum*[T] DSM 14647 genome.

**Locus Tag**	**Gene**	**Predicted Function**
LFTS_00588	*ahpC*	Peroxiredoxin (alkyl hydroperoxide reductase subunit C)
LFTS_02121		
LFTS_02379	*ccmG, dsbE*	Peroxiredoxin
LFTS_02380		
LFTS_01366		
LFTS_00334	*bcp*	Thiol peroxiredoxin Q/BCP
LFTS_00463		
LFTS_00141		Peroxiredoxin family protein
LFTS_00956		
LFTS_00464	*efeB*	Putative iron-dependent peroxidase
LFTS_02122		Cytochrome *c* peroxidase
LFTS_02123	*perR*	Fur family transcriptional regulator peroxide stress response regulator
LFTS_00101		
LFTS_00834		
LFTS_00361		Rubrerythrin
LFTS_01356	*gpmM*	2,3-bisphosphoglycerate-independent phosphoglycerate mutase
LFTS_00965	*icd*	isocitrate dehydrogenase (NAD)<br>Putative isocitrate dehydrogenase (NADP)
LFTS_00966		
LFTS_00293	*degP*	Serine protease Do
LFTS_00852	*trx*	Thioredoxin 1<br>Thioredoxin 2
LFTS_00318		
LFTS_02417	*trxB*	Thioredoxin reductase (NADPH)
LFTS_00473		Glutaredoxin
LFTS_00823		
LFTS_01556	*fumC*	Fumarase C
LFTS_00831		Thiol-disulfide isomerase or thioredoxin
LFTS_00175		
LFTS_01366		
LFTS_02519		
LFTS_01154		Thioredoxin domain-containing protein
LFTS_01773		putative peroxiredoxin
LFTS_00737	*ybbN*	ybbN; DnaK co-chaperone, thioredoxin-like protein
LFTS_00348		L-ectoine synthase
LFTS_00759		High-affinity iron transporter
LFTS_00856		Spermidine synthase
LFTS_00857		S-adenosylmethionine decarboxylase

LFTS_00955		Cobalamin-5'-phosphate synthase
LFTS_01989		Glutathionylspermidine synthase preATP-grasp

TABLE S6 Chemotaxis and motility genes identified in the *Leptospirillium ferriphilum*^{T} DSM 14647 genome.

Locus Tag	Gene	Predicted Function
**Chemotaxis**		
LFTS_00183<br>LFTS_00227<br>LFTS_01445<br>LFTS_01455<br>LFTS_01548<br>LFTS_01731<br>LFTS_02147		methyl-accepting chemotaxis protein
LFTS_00223	*cheR*	chemotaxis protein methyltransferase
LFTS_00224	*cheY3*	two-component systemchemotaxis familyresponse regulator
LFTS_00225	*cheA*	two-component systemchemotaxis familysensor kinase
LFTS_00226	*cheB*	two-component systemchemotaxis familyresponse regulator
LFTS_00228	*cheW*	purine-binding chemotaxis protein
LFTS_00229	*motA*	chemotaxis protein
LFTS_00230	*motB1*	chemotaxis protein
LFTS_00710	*cheZ*	chemotaxis protein
LFTS_00799	*cheV*	two-component systemchemotaxis familyresponse regulator
LFTS_02318	*fliM*	flagellar motor switch protein
Motility		
LFTS_00196	*fleQ*	sigma-54 specific transcriptional regulator, flagellar regulatory protein A
LFTS_00198	*flrB*	two-component systemsensor histidine kinase
LFTS_00199	*flrC*	two-component systemresponse regulator
LFTS_00200	*flgB*	flagellar basal-body rod protein
LFTS_00201	*flgC*	flagellar basal-body rod protein
LFTS_00202	*fliE*	flagellar hook-basal body complex protein
LFTS_00203	*fliF*	flagellar M-ring protein
LFTS_00204	*fliG*	flagellar motor switch protein
LFTS_00205	*fliH*	putative flagellar assembly protein
LFTS_00206	*fliI/YscN*	type III secretion system ATPase
LFTS_00207	*fliJ*	flagellar export protein
LFTS_00209	*fliK*	hook-length control protein
LFTS_00210	*flgD*	flagellar basal-body rod modification protein
LFTS_00211	*flgE*	flagellar hook protein
LFTS_00212	*fliL*	flagellar protein
LFTS_00213	*fliN/fliY*	flagellar motor switch protein

LFTS_00214	*fliO/fliY*	flagellar protein
LFTS_00215	*fliP*	flagellar biosynthetic protein
LFTS_00216	*fliQ*	flagellar biosynthetic protein
LFTS_00217	*fliR*	flagellar biosynthetic protein
LFTS_00218	*flhB*	flagellar biosynthetic protein
LFTS_00219	*flhA*	flagellar biosynthesis protein
LFTS_00220	*flhF*	flagellar biosynthesis protein
LFTS_00221	*flhG*	flagellar biosynthesis protein
LFTS_00222	*fliA*	RNA polymerase sigma factor for flagellar operon
LFTS_02334	*fliS*	flagellar protein
LFTS_02335	*fliD*	flagellar hook-associated protein 2
LFTS_02336	*fliC1*	flagellin
LFTS_02337	*fliW*	flagellar assembly factor
LFTS_02339	*flgL*	flagellar hook-associated protein
LFTS_02340	*flgK*	flagellar hook-associated protein
LFTS_02341	*flgN*	export chaperone for FlgK and FlgL
LFTS_02342	*flgM*	anti-sigma-28 factor
LFTS_02343	*fliC2*	flagellin
LFTS_02344	*flgJ*	flagellar protein
LFTS_02345	*flgI*	flagellar P-ring protein precursor
LFTS_02346	*flgH*	flagellar L-ring protein precursor
LFTS_02347	*flgA*	flagella basal body P-ring formation protein
LFTS_02348	*flgG*	flagellar basal-body rod protein
LFTS_02349	*flgE*	flagellar basal-body rod protein

TABLE S7 Quorum sensing and c-di-GMP genes identified in the *Leptospirillium ferriphilum*^{T} DSM 14647 genome.

Locus Tag	Gene	Predicted Function
**Quorum sensing**		
LFTS_00514	*rpfF*	DSF synthase
LFTS_00515	*rpfC*	Hpt domain-containing protein
LFTS_00516	*rpfC*	Signal transduction histidine kinase
LFTS_00517	*rpfG*	Two-component system response regulator
LFTS_00133		Regulatory protein luxR family
LFTS_00589	*sdiA*	Quorum-sensing transcriptional activator; LuxR family transcriptional regulator
LFTS_01215		Autoinducer binding domain
LFTS_01241	*sdiA*	LuxR family transcriptional regulator
**c-di-GMP specific phosphodiestereases (EAL domain-containing proteins)**		
LFTS_00034<br>LFTS_00314	*gmr, csgD*	EAL domain, c-di-GMP-specific phosphodiesterase class I (or its enzymatically inactive variant)
**Genes encoding proteins with EAL and GGDEF domains**		
LFTS_00089		Diguanylate cyclase / phosphodiesterase with GAF sensor
LFTS_00405		PAS/PAC and Chase sensor-containing diguanylate cyclase/phosphodiesterase
LFTS_00479<br>LFTS_00575<br>LFTS_00733		Signalling repeat/GGDEF domain/EAL domain protein
LFTS_00493<br>LFTS_00663<br>LFTS_00709<br>LFTS_01908		Diguanylate cyclase/phosphodiesterase with PAS/PAC sensor(S)
LFTS_00968		Diguanylate cyclase/phosphodiesterase with GAF sensor<br>Diguanylate cyclase/phosphodiesterase (GGDEF & EAL domains)
LFTS_01006		Diguanylate cyclase/phosphodiesterase (GGDEF & EAL domains)
LFTS_01595		Response regulator receiver modulated diguanylate cyclase/phosphodiesterase with PAS/PAC sensor(S)
**Putative diguanylate cyclases (GGDEF domain-containing proteins)**		
LFTS_00174<br>LFTS_00501<br>LFTS_00511<br>LFTS_00714<br>LFTS_00872<br>LFTS_01061<br>LFTS_02076	*dosC*	Diguanylate cyclase; cold- and stationary phase-induced oxygen-dependent biofilm regulator
LFTS_00029<br>LFTS_00503<br>LFTS_00562<br>LFTS_00858<br>LFTS_01493		GGDEF family protein, diguanylate cyclase

LFTS_01845<br>LFTS_02591		
LFTS_00306<br>LFTS_01577		Response regulator receiver modulated diguanylate cyclase
LFTS_00510		PAS domain S-box-containing protein/diguanylate cyclase (GGDEF) domain-containing protein
**c-di-GMP effector proteins**		
LFTS_00568<br>LFTS_00573<br>LFTS_02308		PilZ domain-containing protein
LFTS_00504	*popA*	two-component system, cell cycle response regulator

TABLE S8 Biofilm formation genes identified in the *Leptospirillium ferriphilum*^{T} DSM 14647 genome.

**Locus Tag**	**Gene**	**Predicted Function**
**Cellulose production**		
LFTS_00736	*bcsQ*	Cellulose biosynthesis protein
LFTS_00738	*bcsB*	Cellulose synthase subunit
LFTS_00739	*bcsA*	Cellulose synthase (UDP-forming)
LFTS_02306	*rexA*	Cellulase family 8, oligosaccharide reducing-end xylanase
LFTS_02307	*bcsB*	Cellulose synthase subunit
LFTS_02308	*bcsA*	Cellulose synthase (UDP-forming) PilZ domain-containing protein
LFTS_02310	*bcsA*	Cellulose synthase (UDP-forming)
LFTS_02311	*bcsC*	Probable cellulose synthase operon protein C
**putative pel operon**		
LFTS_01242	*pelG*	Putative membrane protein
LFTS_01243	*pelF*	Pellicle/biofilm biosynthesis glycosyltransferase
LFTS_01244	*mpeU (pelE)*	Bilin biosynthesis protein
LFTS_01245	*pelD*	Response regulator receiver modulated diguanylate cyclase
LFTS_01246		Glycoside-hydrolase family GH114
LFTS_01247	*lpoB (pelC)*	Outer membrane lipoprotein
LFTS_01248		Putative PEP-CTERM system tetratricopeptide repeat lipoprotein
LFTS_01249		Putative PEP-CTERM system tetratricopeptide repeat lipoprotein
LFTS_01250	*pelA*	Putative membrane protein-like protein, polysaccharide deacetylase
**Lipopolysaccharide synthesis**		
LFTS_00080	*lkxH*	UDP-2,3-diacylglucosamine pyrophosphatase
LFTS_00081		1,2-diacylglycerol 3-beta-galactosyltransferase
LFTS_01292	*lpxD*	UDP-3-O-[3-hydroxymyristoyl] glucosamine N-acyltransferase
LFTS_01293	*fabZ*	3R-hydroxymyristoyl-[acyl-carrier-protein] dehydratase
LFTS_01294	*lpxA*	UDP-N-acetylglucosamine O-acyltransferase
LFTS_01297	*lpxB*	Lipid-A-disaccharide synthase
LFTS_01299	*msbA*	Fused lipid transporter subunits of ABC superfamily: membrane component/ATP-binding cassette
LFTS_01300	*kdtA, waaA*	3-deoxy-D-manno-octulosonic-acid transferase
LFTS_01301	*lpxK*	Lipid A tetraacyldisaccharide 4'-kinase
LFTS_01302	*lpxL*	Lauryl-acyl carrier protein (ACP)-dependent
LFTS_01303	*rfaF*	ADP-heptose:LPS heptosyltransferase II
LFTS_01304	*rfaC*	ADP-heptose:LPS heptosyl transferase I
LFTS_01305	*rfaC*	ADP-heptose:LPS heptosyl transferase I

LFTS_01306	*kdtX*	Lipopolysaccharide core biosynthesis glycosyl transferase
LFTS_01307	*wbdM*	Glycosyltransferase
LFTS_01298<br>LFTS_01308<br>LFTS_01310		Glycosyl transferase family protein
LFTS_01311	*rfaF*	ADP-heptose:LPS heptosyltransferase II
LFTS_01312	*gmhB*	D,D-heptose 1,7-bisphosphate phosphatase
LFTS_01314	*rfaF*	ADP-heptose:LPS heptosyltransferase II
LFTS_01315	*rfaQ*	Lipopolysaccharide core biosynthesis protein
LFTS_01316	*pgaB*	Biofilm adhesin polysaccharide PGA export lipoprotein with a polysaccharide deacetylase activity needed for export
LFTS_01317	*pimB*	Group 1 glycosyl transferase
LFTS_01323	*rfaL*	O-antigen ligase
LFTS_01328	*arnT*	4-amino-4-deoxy-L-arabinose transferase
LFTS_01329	*rfaF*	ADP-heptose:LPS heptosyltransferase II
LFTS_01330	*DPM1*	Dolichol-phosphate mannosyltransferase
LFTS_01331	*arnT*	4-amino-4-deoxy-L-arabinose transferase
LFTS_01345	*lptG*	Lipopolysaccharide export ABC permease of the LptBFGC export complex
LFTS_01346	*lptF*	Lipopolysaccharide export ABC permease of the LptBFGC export complex
LFTS_01629	*rfaD*	ADP-L-glycero-D-mannoheptose-6-epimerase
LFTS_01631	*mef*	Macrolide-efflux protein
LFTS_01632	*BGLUT*	UDP-glucose:tetrahydrobiopterin glucosyltransferase
LFTS_01633	*otsA*	Trehalose 6-phosphate synthase
LFTS_01634	*otsB*	Trehalose 6-phosphate phosphatase
LFTS_01635	*rfbD, rmlD*	dTDP-4-dehydrorhamnose reductase
LFTS_01721	*arnE*	Undecaprenyl phosphate-alpha-L-ara4N exporter
LFTS_01782	*hddC*	D-glycero-alpha-D-manno-heptose 1-phosphate guanylyltransferase
LFTS_01783	*galE*	UDP-galactose-4-epimerase
LFTS_01808	*rfaF*	ADP-heptose:LPS heptosyltransferase
LFTS_01809	*mtfB*	Mannosyltransferase, alpha-1,3-rhamnosyltransferase
LFTS_01810		Putative group 1 glycosyltransferase
LFTS_01811	*wbpX*	Glycosyltransferase, alpha-1,2-rhamnosyltransferase
LFTS_01812	*ABC-2.LPSE.A*	Lipopolysaccharide transport system ATP-binding protein
LFTS_01813	*ABC-2.LPSE.P*	Lipopolysaccharide transport system permease protein
LFTS_01814	*rfbP*	Undecaprenyl-phosphate galactose phosphotransferase
LFTS_01815	*pimB*	Group 1 glycosyl transferase, phosphatidylinositol alpha-1,6-mannosyltransferase
LFTS_01816	*mtfB*	Mannosyltransferase, alpha-1,3-rhamnosyltransferase

LFTS_02328	*rfaF*	ADP-heptose:LPS heptosyltransferase II
LFTS_02329	*cgeB*	Spore maturation protein
LFTS_02330	*rfaF*	ADP-heptose:LPS heptosyltransferase II
LFTS_02331	*tam*	Putative trans-aconitate 2-methyltransferase
LFTS_02332	*cgeB*	Spore maturation protein
LFTS_02488	*lpxC*	UDP-3-O-[3-hydroxymyristoyl] N-acetylglucosamine deacetylase
**Extracellular polysaccharide production and export**		
LFTS_00424	*wzzE*	Polysaccharide chain length modulation protein
LFTS_00488	*rfbB, rffG*	dTDP-glucose 4,6-dehydratase
LFTS_00489<br>LFTS_00121	*rfbA, rffA*	Glucose-1-phosphate thymidylyltransferase
LFTS_00490	*rfbC,rmlC*	dTDP-4-dehydrorhamnose 3,5-epimerase
LFTS_00492	*(algK)*	Sel-1 repeat protein
LFTS_00528	*pimA*	Phosphatidylinositol alpha-mannosyltransferase
LFTS_00529<br>LFTS_00530<br>LFTS_00531<br>LFTS_00532<br>LFTS_00540		Glycosyl transferase family protein
LFTS_00535		EpsI family protein
LFTS_00536	*epsH*	Eight transmembrane protein, glycosyltransferase
LFTS_00537	*rfbA, rffA*	Glucose-1-phosphate thymidylyltransferase
LFTS_00538		Putative PEP-CTERM system histidine kinase
LFTS_00539		Putative PEP-CTERM system histidine kinase
LFTS_00541	*wbdM*	Glycosyltransferase
LFTS_00543		Putative inner membrane protein, O-antigen polymerase, Exopolysaccharide biosynthesis family protein
LFTS_00544<br>LFTS_00545	*rfaG*	Glucosyltransferase I
LFTS_00546	*pimB*	Group 1 glycosyl transferase, phosphatidylinositol alpha-1,6-mannosyltransferase
LFTS_00547	*pgaC, icaA*	Predicted glycosyl transferase, biofilm PGA synthesis N-glycosyltransferase
LFTS_00548	*pgaB*	Biofilm adhesin polysaccharide PGA export lipoprotein with a polysaccharide deacetylase activity needed for export
LFTS_00550	*wza*	Polysaccharide export outer membrane protein
LFTS_00551	*wcaJ*	Putative colanic acid biosysnthesis UDP-glucose lipid carrier transferase
LFTS_00552	*wzz*	Chain length determinant protein
LFTS_00565	*glf*	UDP-galactopyranose mutase
LFTS_00566		UDP-glucuronate 4-epimerase
LFTS_00568		PilZ domain-containing protein, sigma 54-dependent response regulator
LFTS_00569	*glmS*	Glucosamine--fructose-6-phosphate aminotransferase

LFTS_00570	*wbpA*	UDP-N-acetyl-D-glucosamine dehydrogenase
LFTS_00572<br>LFTS_00683<br>LFTS_00656	*galE*	UDP-galactose-4-epimerase
LFTS_00573		PilZ domain-containing protein, sigma 54-dependent response regulator
LFTS_00769	*pmm, pgm*	Phosphomannomutase / phosphoglucomutase
LFTS_00770	*algA, xanB, rfbA, wbpW, pslB*	Mannose-1-phosphate guanylyltransferase / mannose-6-phosphate isomerase
LFTS_01394	*pgm*	Phosphoglucomutase
LFTS_01526	*uxs 1*	UDP-glucuronate decarboxylase 1
LFTS_01528	*ugd*	UDP-glucose 6-dehydrogenase
LFTS_01564		Membrane protein; Chitin synthase
LFTS_02426	*galT*	Galactose-1-phosphate uridylyltransferase

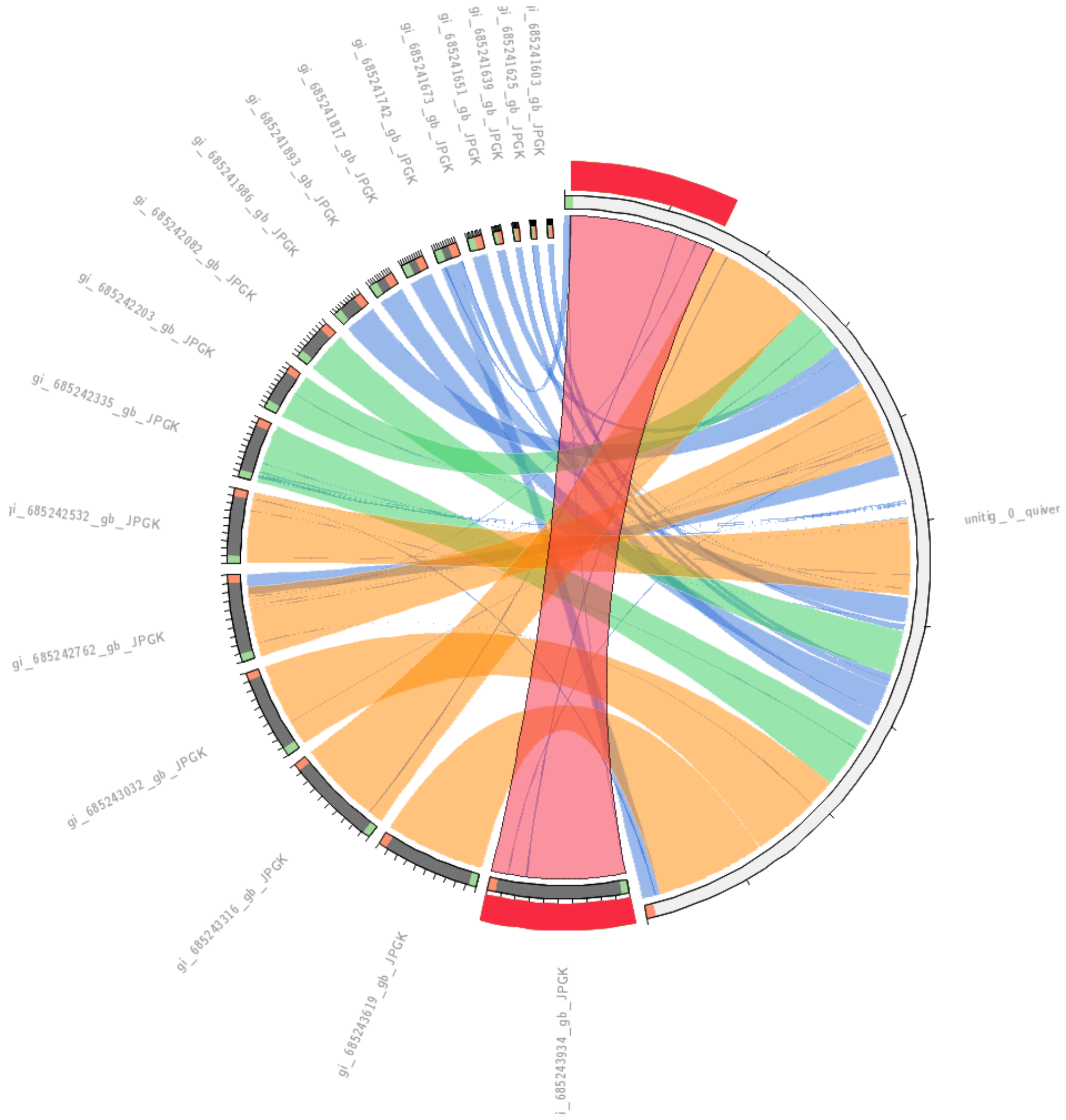

FIG S1 Circoletto plot (1) (http://tools.bat.infspire.org/circoletto/ 10/01/2015) showing a comparison between the new assembly contig1 (white) and the 18 contigs of the draft genome (2). Default settings were used and colored ribbons indicate relative alignment scores which in this case primarily reflect sequence length.

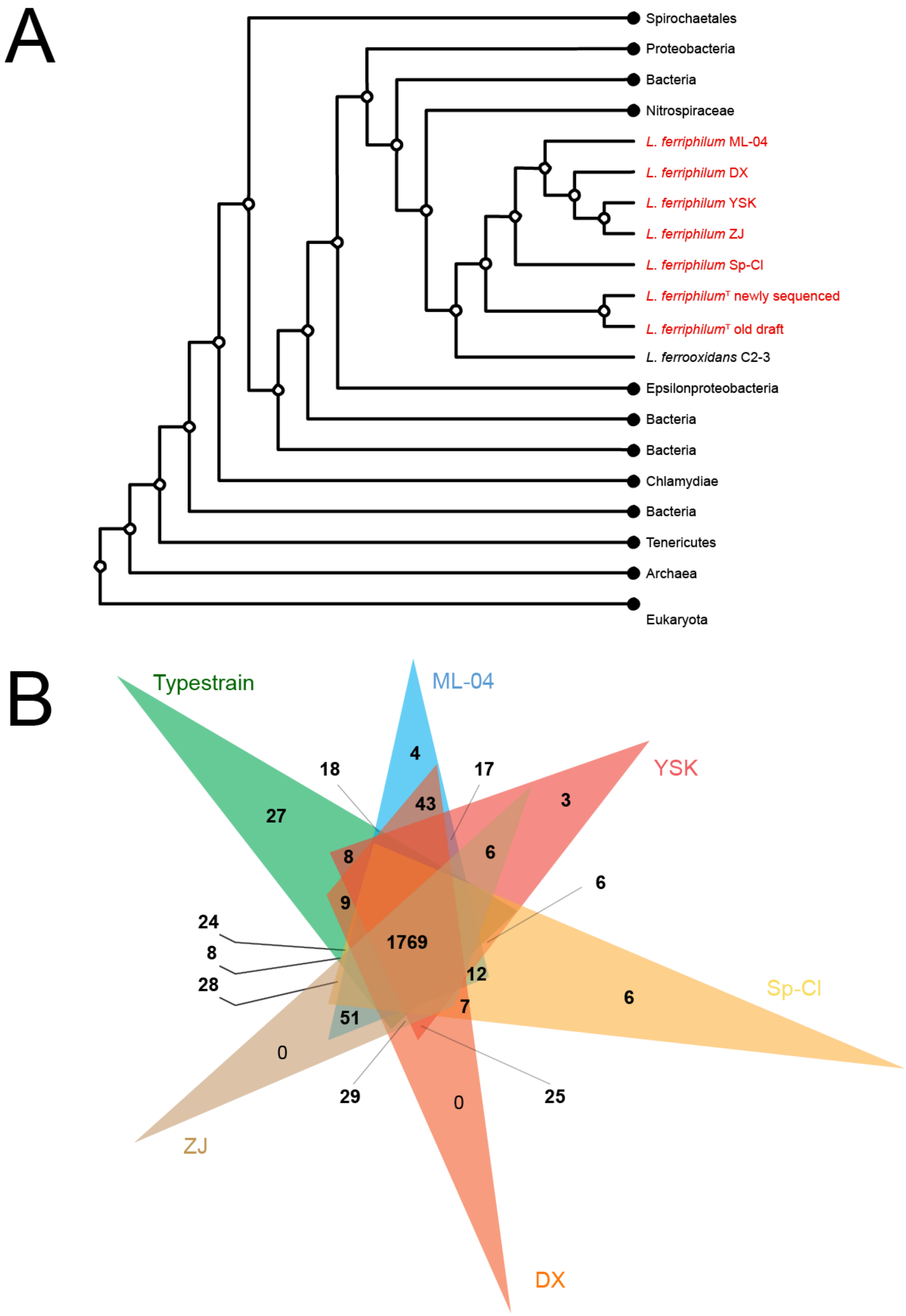
A
Spirochaetales
Proteobacteria
Bacteria
Nitrospiraceae
L. ferriphilum ML-04
L. ferriphilum DX
L. ferriphilum YSK
L. ferriphilum ZJ
L. ferriphilum Sp-Cl
L. ferriphilumT newly sequenced
L. ferriphilumT old draft
L. ferrooxidans C2-3
Epsilonproteobacteria
Bacteria
Bacteria
Chlamydiae
Bacteria
Tenericutes
Archaea
Eukaryota
B
Typestrain
ML-04
YSK
Sp-Cl
DX
ZJ
4
18
17
27
43
3
8
6
6
9
24
1769
8
12
28
7
6
51
0
29
0
25

FIG S2 Phylogenetic placement and relationship of *L. ferriphilum* strains. Shown are an evolutionary tree of the available strains (A), and a Venn diagram of orthologous gene clusters (B). The phylogenetic tree was constructed with CVTree3 (3) (http://tlife.fudan.edu.cn/archaea/cvtree/cvtree3/ 30/10/2017) using amino acid sequences with a K-tuple length of 6. Venn diagram was created with OrthoVenn (4) (http://www.bioinfogenome.net/OrthoVenn/index.php 30/10/2017) using protein sequences downloaded from NCBI as input. More detailed analysis as well as functional categories assigned to shared genes can be found under url http://www.bioinfogenome.net/OrthoVenn/result.php?ID=d0faeba113424e41958bda82bce23f37.

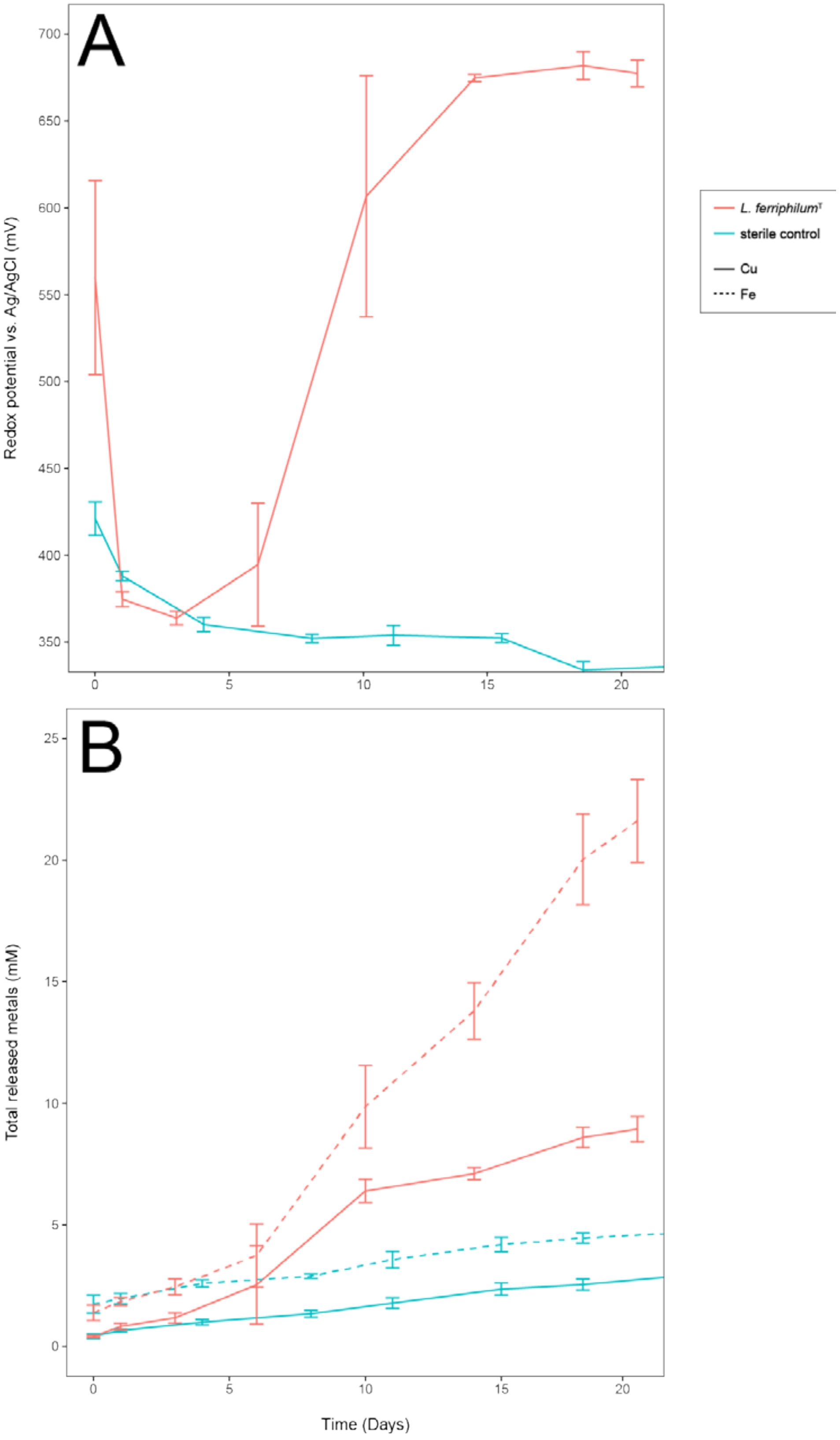

FIG S3 Redox potential (A) and release of copper and iron (B) from chalcopyrite concentrate during bioleaching by *L. ferriphilum*^{T} compared to a sterile control.

REPORT S1 Details of the PacBio sequencing run.

Reports for Job pb_238_HGAP3_CTR

SMRT Cells: 2 Movies: 2

Overview

Job Metric	Value
Polished Contigs	2
Adapter Dimers (0-10bp)	0.0%
Short Inserts (11-100bp)	0.0%
Number of Bases	1,686,331,902
Number of Reads	136,837
N50 Read Length	17,183
Mean Read Length	12,323
Mean Read Score	0.86
Mapped Reads	128,992
Mapped Read Length of Insert	8,775
Average Reference Length	1,305,184
Average Reference Bases Called	100.0%
Average Reference Consensus Concordance	99.99%
Average Reference Coverage	565.78

Adapters

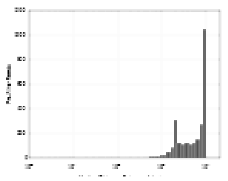

Observed Insert Length Distribution Histogram

Subread Filtering

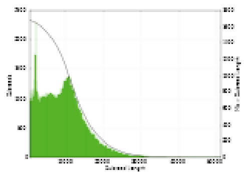

Subread Filtering

Mapping

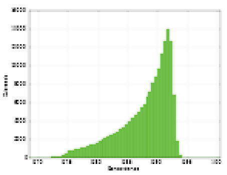

Mapped Subread Concordance

Mapping

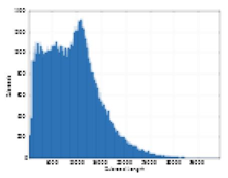

Mapped Subread Length

Mapping

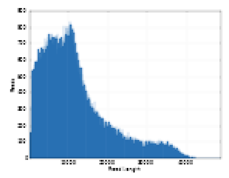

Mapped Polymerase Read Length

Coverage

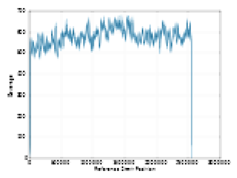

Coverage Across Reference

Coverage

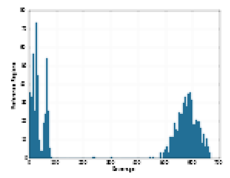

Depth of Coverage

Corrections

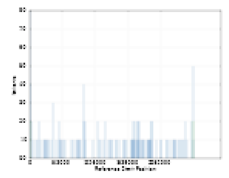

Corrections Across Contigs

Filtering

Filtering

Metrics	Pre-Filter	Post-Filter
Polymerase Read Bases	1861631140	1686331902
Polymerase Reads	300584	136837
Polymerase Read N50	16709	17183
Polymerase Read Length	6193	12323
Polymerase Read Quality	0.436	0.862

Polymerase Read Length

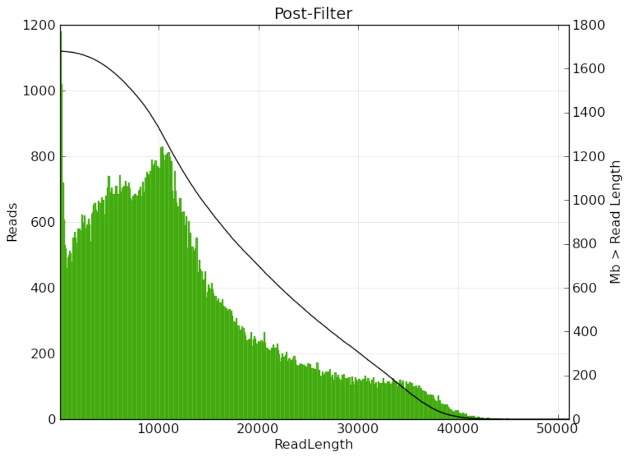

Polymerase Read Quality

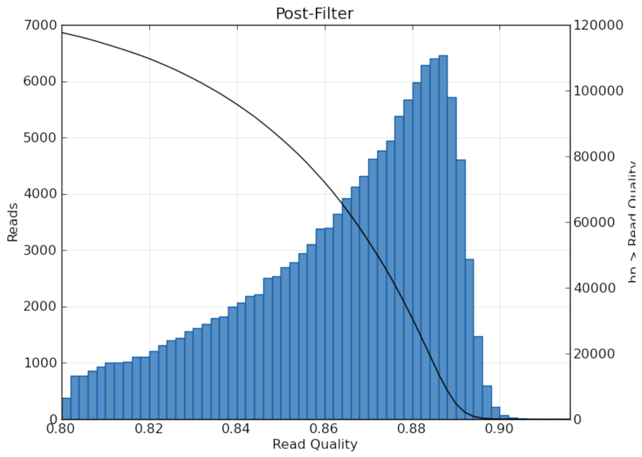

Subread Filtering

Mean Subread length	9,262	N50	12,114
Total Number of Bases	1,682,372,470	Number of Reads	181,637

Subread Filtering

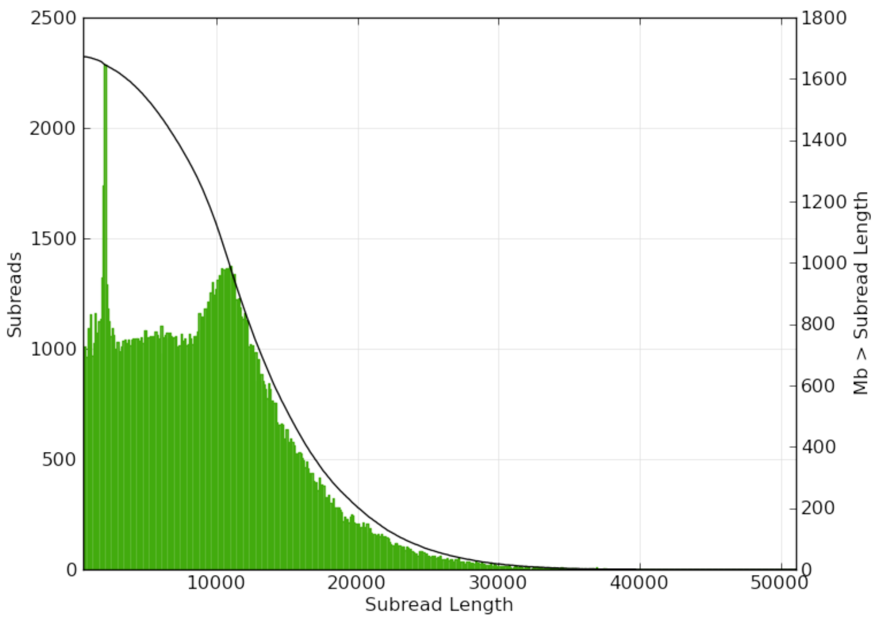

Adapters

Adapter Dimers (0-10bp)	0.0%
Short Inserts (11-100bp)	0.0%

Observed Insert Length Distribution

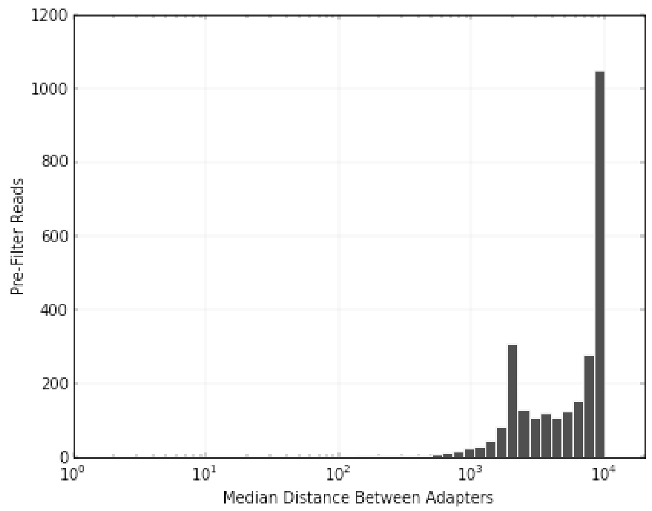

Loading

SMRT Cell ID	Productive ZMWs	ZMW Loading For Productivity 0	ZMW Loading For Productivity 1	ZMW Loading For Productivity 2
m150926_080424_42237_c100887892550000001823197504021641	150,292	32.84%	53.96%	13.2%
m150926_034511_42237_c100887892550000001823197504021640	150,292	45.69%	44.41%	9.9%

Mapping

Mapped Subread Length N50 (bp)	11,696	Mapped Polymerase Read Length 95% (bp)	31,070
Mapped Subread Length Mean (bp)	8,955	Mapped Polymerase Read Length Max (bp)	48,549

Mapping Stats Summary

Movie	Mapped Read	Mapped Polymerase Read Length	Mapped Polymerase Read Length N50	Mapped Subreads	Mapped Subread Bases	Mapped Subread Length	Mean Mapped Subread Concordance
All Movies	128,992	11,821	16038	169,430	1517325035	8,955	0.872
m150926_080424_42237_c100887892550000001823197504021641_s1_p0	70,296	11,851	16028	92,705	828324597	8,935	0.870
m150926_034511_42237_c100887892550000001823197504021640_s1_p0	58,696	11,786	16052	76,725	689000438	8,980	0.875

Mapped Subread Concordance

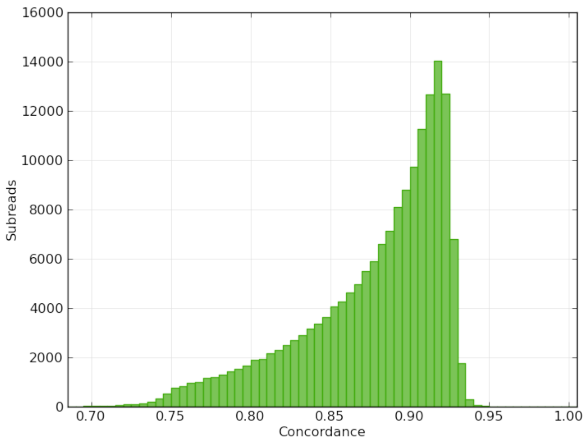

Mapped Polymerase Read Length

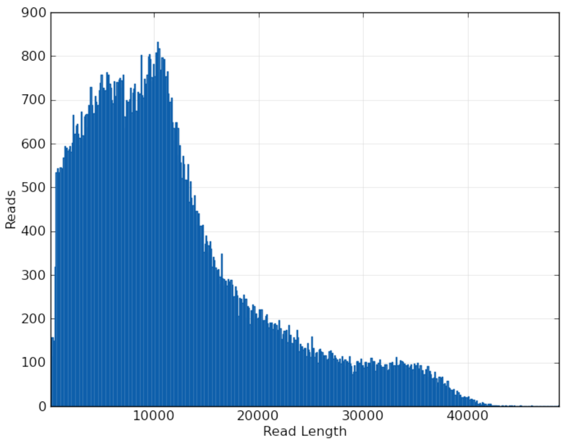

Mapped Subread Length

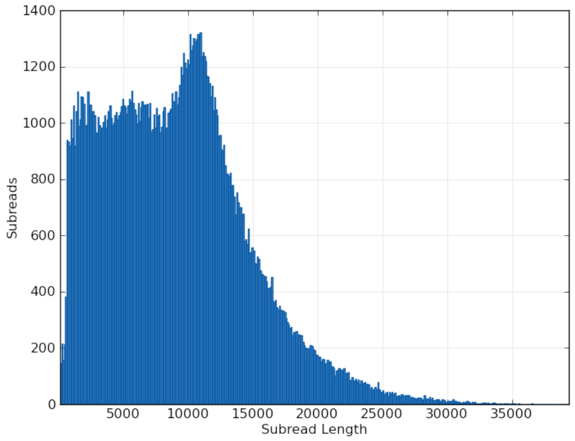

Coverage

Mean Coverage	565.78
Missing Bases (%)	0.0

Coverage Across Reference

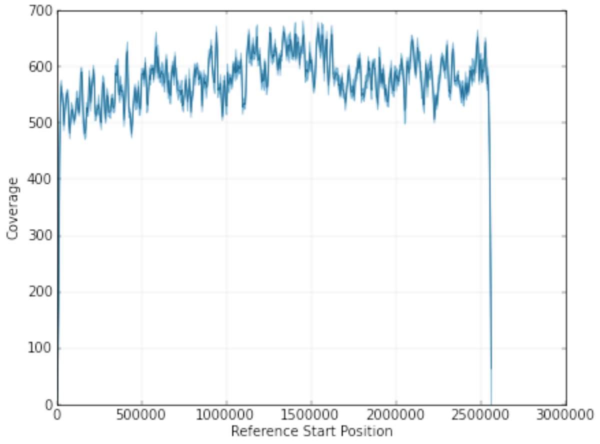

Observed depth of coverage across unitig_0 (window size = 5008bp).

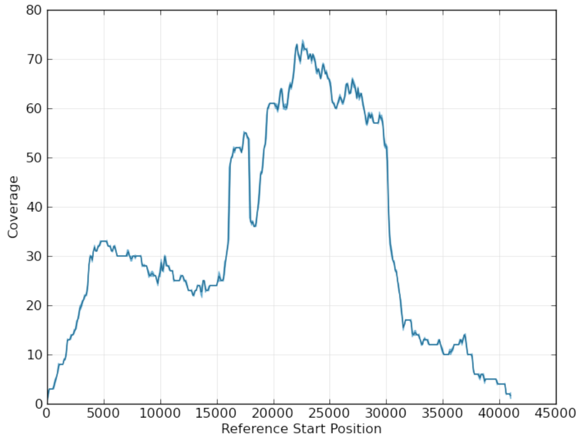

Observed depth of coverage across unitig_1 (window size = 100bp).

Depth Of Coverage

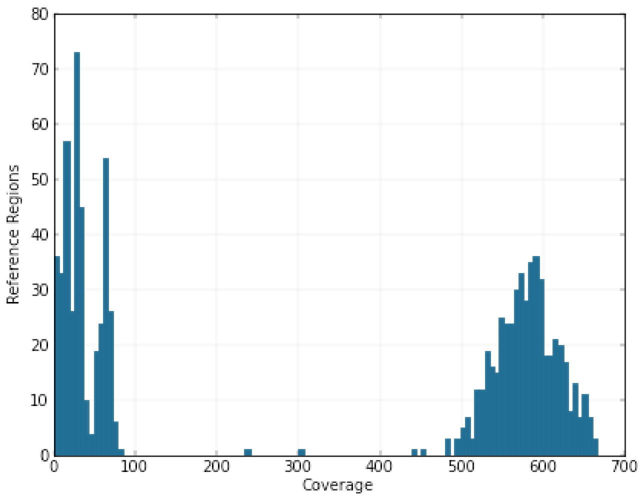

Depth of coverage distribution

Pre-Assembler Report

Polymerase Read Bases	1,682,372,470	Length Cutoff	23,786
Seed Bases	90,002,907	Pre-Assembled bases	70,524,771
Pre-Assembled Yield	.784	Pre-Assembled Reads	3,720
Pre-Assembled Reads Length	18,958	Pre-Assembled N50	24,656

Polished Assembly

Polished Contigs	2	Max Contig Length	2,569,357
N50 Contig Length	2,569,357	Sum of Contig Lengths	2,610,531

Contig Coverage Vs Confidence

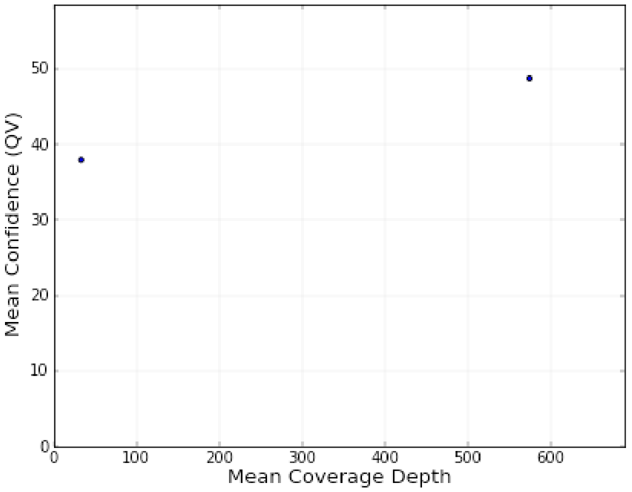

NOTE: Confidence is estimated by Quiver based on internal read consistency, similar to how phred scores are traditionally computed.

Top Corrections

Sequence	Position	Correction	Type	Coverage	Confidence	Genotype
unitig_0	2,401,913	2401913_2401914insG	INS	100	53	haploid
unitig_0	5,786	5786_5787insT	INS	38	52	haploid
unitig_0	110,087	110087_110088insG	INS	100	52	haploid
unitig_0	1,469,353	1469353_1469354insG	INS	100	52	haploid
unitig_0	252,057	252057_252058insG	INS	100	51	haploid
unitig_0	481,852	481852_481853insG	INS	100	51	haploid
unitig_0	783,126	783126_783127insC	INS	100	51	haploid
unitig_0	1,099,462	1099462_1099463insG	INS	100	51	haploid
unitig_0	1,152,713	1152713_1152714insC	INS	100	51	haploid
unitig_0	1,602,548	1602548_1602549insG	INS	100	51	haploid
unitig_0	1,873,482	1873482_1873483insG	INS	100	51	haploid
unitig_0	1,889,871	1889871_1889872insC	INS	100	51	haploid
unitig_0	2,363,046	2363046_2363047insG	INS	100	51	haploid

unitig_0	2,564,819	2564819_2564820insC	INS	14	51	haploid
unitig_1	4,411	4411_4412insT	INS	30	51	haploid
unitig_1	8,659	8659_8660insT	INS	28	51	haploid
unitig_1	14,554	14554_14555insT	INS	24	51	haploid
unitig_1	14,613	14613_14614insTTCTC	INS	24	51	haploid
unitig_1	14,688	14688_14689insC	INS	24	51	haploid
unitig_1	14,807	14807_14808insT	INS	24	51	haploid
unitig_1	15,221	15221_15222insCAG	INS	25	51	haploid
unitig_0	1,817	1817_1818insA	INS	11	50	haploid
unitig_0	4,594	4594_4595insC	INS	29	50	haploid
unitig_0	6,607	6607_6608insC	INS	54	50	haploid
unitig_0	17,580	17580_17581insG	INS	100	50	haploid
unitig_0	149,813	149813_149814insG	INS	100	50	haploid
unitig_0	256,607	256607_256608insC	INS	100	50	haploid
unitig_0	293,067	293067_293068insC	INS	100	50	haploid
unitig_0	366,710	366710_366711insG	INS	100	50	haploid
unitig_0	451,437	451437_451438insC	INS	100	50	haploid
unitig_0	466,869	466869_466870insC	INS	100	50	haploid
unitig_0	654,441	654441_654442insG	INS	100	50	haploid
unitig_0	671,519	671519_671520insC	INS	100	50	haploid
unitig_0	846,989	846989_846990insG	INS	100	50	haploid
unitig_0	857,372	857372_857373insC	INS	100	50	haploid
unitig_0	1,115,970	1115970_1115971insC	INS	100	50	haploid
unitig_0	1,192,088	1192088_1192089insC	INS	100	50	haploid
unitig_0	1,278,328	1278328_1278329insC	INS	100	50	haploid
unitig_0	1,353,258	1353258_1353259insC	INS	100	50	haploid
unitig_0	1,514,175	1514175_1514176insC	INS	100	50	haploid
unitig_0	1,523,097	1523097_1523098insG	INS	100	50	haploid
unitig_0	1,686,356	1686356_1686357insG	INS	100	50	haploid
unitig_0	1,724,903	1724903_1724904insG	INS	100	50	haploid
unitig_0	1,772,881	1772881_1772882insC	INS	100	50	haploid
unitig_0	1,877,219	1877219_1877220insG	INS	100	50	haploid
unitig_0	1,912,330	1912330_1912331insG	INS	100	50	haploid
unitig_0	1,977,390	1977390_1977391insG	INS	100	50	haploid
unitig_0	2,190,358	2190358_2190359insG	INS	100	50	haploid
unitig_0	2,286,825	2286825_2286826insG	INS	100	50	haploid
unitig_0	2,330,394	2330394_2330395insG	INS	100	50	haploid
unitig_0	2,348,211	2348211_2348212insC	INS	100	50	haploid
unitig_0	2,436,019	2436019_2436020insG	INS	100	50	haploid
unitig_0	2,464,531	2464531_2464532insC	INS	100	50	haploid
unitig_0	2,551,483	2551483_2551484insC	INS	100	50	haploid
unitig_1	1,976	1976_1977insC	INS	12	50	haploid
unitig_1	2,191	2191_2192insA	INS	13	50	haploid
unitig_1	7,083	7083_7084insCC	INS	29	50	haploid
unitig_1	8,577	8577_8578insG	INS	28	50	haploid
unitig_1	8,723	8723_8724insT	INS	27	50	haploid
unitig_1	12,367	12367_12368insC	INS	23	50	haploid
unitig_1	13,826	13826delA	DEL	25	50	haploid
unitig_1	14,218	14218_14219insC	INS	23	50	haploid
unitig_1	14,632	14632delAA	DEL	24	50	haploid
unitig_1	14,916	14916_14917insG	INS	24	50	haploid
unitig_1	15,052	15052_15053insC	INS	24	50	haploid
unitig_1	15,288	15288_15289insT	INS	27	50	haploid
unitig_1	15,438	15438_15439insA	INS	25	50	haploid
unitig_1	15,554	15554_15555insA	INS	25	50	haploid
unitig_0	2,191	2191_2192insG	INS	13	49	haploid
unitig_0	3,740	3740delC	DEL	26	49	haploid
unitig_0	19,193	19193_19194insG	INS	100	49	haploid
unitig_0	49,146	49146_49147insC	INS	100	49	haploid
unitig_0	93,555	93555_93556insG	INS	100	49	haploid
unitig_0	182,764	182764_182765insG	INS	100	49	haploid
unitig_0	210,334	210334_210335insG	INS	100	49	haploid
unitig_0	233,679	233679_233680insG	INS	100	49	haploid
unitig_0	247,181	247181_247182insC	INS	100	49	haploid
unitig_0	366,371	366371_366372insG	INS	100	49	haploid
unitig_0	368,802	368802_368803insG	INS	100	49	haploid
unitig_0	465,391	465391_465392insC	INS	100	49	haploid
unitig_0	475,673	475673_475674insG	INS	100	49	haploid
[illegible]	[illegible]	[illegible]	[illegible]	[illegible]	[illegible]	[illegible]

unitig_0	564,532	564532_564533insC	INS	100	49	haploid
unitig_0	730,933	730933_730934insC	INS	100	49	haploid
unitig_0	759,497	759497_759498insG	INS	100	49	haploid
unitig_0	764,923	764923_764924insG	INS	100	49	haploid
unitig_0	769,268	769268_769269insC	INS	100	49	haploid
unitig_0	811,375	811375_811376insG	INS	100	49	haploid
unitig_0	867,219	867219_867220insG	INS	100	49	haploid
unitig_0	868,383	868383_868384insC	INS	100	49	haploid
unitig_0	880,064	880064_880065insG	INS	100	49	haploid
unitig_0	982,668	982668_982669insC	INS	100	49	haploid
unitig_0	1,229,785	1229785_1229786insC	INS	100	49	haploid
unitig_0	1,259,693	1259693_1259694insG	INS	100	49	haploid
unitig_0	1,288,176	1288176_1288177insC	INS	100	49	haploid
unitig_0	1,410,660	1410660_1410661insC	INS	100	49	haploid
unitig_0	1,619,692	1619692_1619693insC	INS	100	49	haploid
unitig_0	1,624,878	1624878_1624879insG	INS	100	49	haploid
unitig_0	1,627,765	1627765_1627766insC	INS	100	49	haploid
unitig_0	1,692,837	1692837_1692838insG	INS	100	49	haploid
unitig_0	1,699,835	1699835_1699836insC	INS	100	49	haploid

Corrections

Consensus Calling Results

Reference	Reference Length	Bases Called	Consensus Concordance	Coverage
unitig_0	2,569,226	100.0%	99.9947%	574.31
unitig_1	41,141	99.93%	99.8735%	32.91

Corrections Across Reference

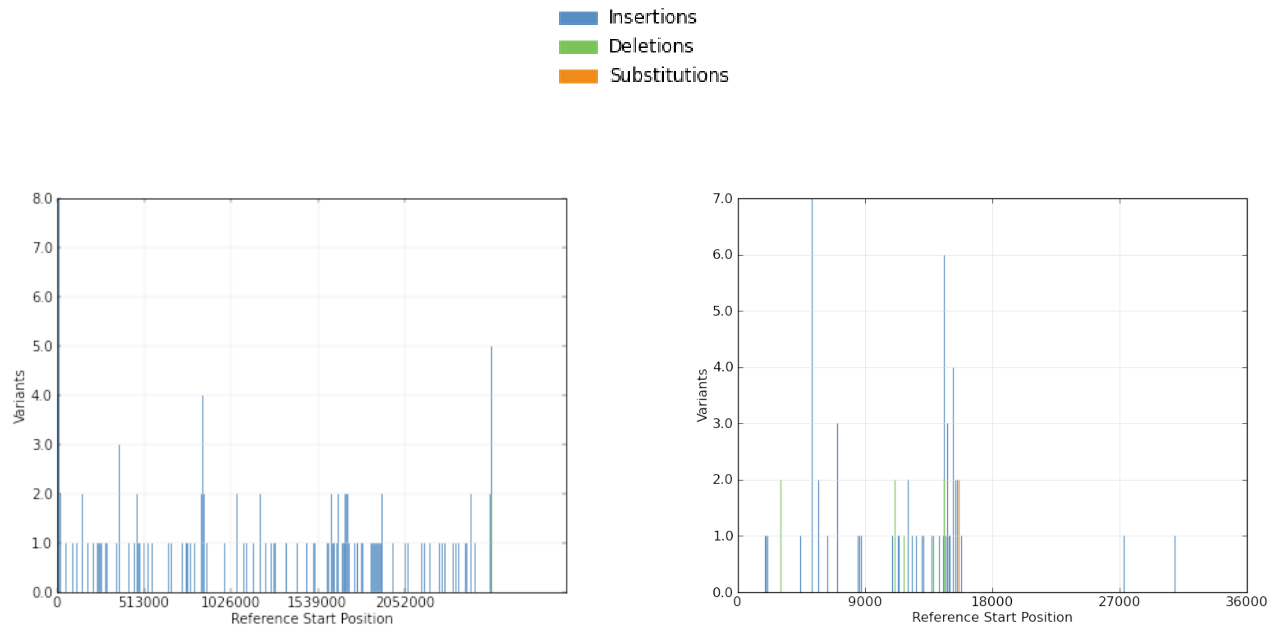

Observed variants across unitig_0.

Observed variants across unitig_1.

Generated by SMRT® Portal. Wed Sep 30 10:56:02 CEST 2015
For Research Use Only. Not for use in diagnostic procedures.

REPORT S2 Summary of CRISPR and phage association predictions for contig1 and contig2.

CRISPR Analysis of the two assembly contigs

Two assembly contigs unitq_0 (chromosome sequence of Leptospirillum ferriphilum) and unitiq_1 were analyzed.

Prediction with CRISPRone

http://omics.informatics.indiana.edu/CRISPRone/show-col.php?col=tmp&id=bT65HI1U

metaCRT

http://omics.informatics.indiana.edu/CRISPR/

Used for CRISPR detection and extraction of repeat and spacer sequences

Comparison of spacer sequences to unitiq_1 with blast:

qseqid	sseqid	pident	length	mismatch	gapopen	qstart	qend	qlen	qcovs	sstart	send	slen	evalue	bitscore
metacrt_contigs-mgmt_unitig_0_quiver:c1:p1162280	unitig_1_quiver	75.00	24	6	0	9	32	33	73	40285	40308	41174	0.34	20.6
metacrt_contigs-mgmt_unitig_0_quiver:c1:p1162341	unitig_1_quiver	77.27	22	5	0	1	22	33	91	5125	5146	41174	0.34	20.6
metacrt_contigs-mgmt_unitig_0_quiver:c1:p1162341	unitig_1_quiver	77.27	22	5	0	9	30	33	91	13922	13901	41174	0.34	20.6
metacrt_contigs-mgmt_unitig_0_quiver:c1:p1162402	unitig_1_quiver	92.86	14	1	0	6	19	33	42	11374	11387	41174	0.34	20.6
metacrt_contigs-mgmt_unitig_0_quiver:c1:p1162463	unitig_1_quiver	88.89	18	2	0	11	28	33	67	9410	9427	41174	0.037	23.8
metacrt_contigs-mgmt_unitig_0_quiver:c1:p1162463	unitig_1_quiver	77.27	22	5	0	11	32	33	67	40729	40708	41174	0.34	20.6
metacrt_contigs-mgmt_unitig_0_quiver:c1:p1162585	unitig_1_quiver	100.00	12	0	0	17	28	32	38	11330	11319	41174	0.34	20.6
metacrt_contigs-mgmt_unitig_0_quiver:c1:p1162645	unitig_1_quiver	100.00	12	0	0	12	23	33	36	25856	25845	41174	0.34	20.6
metacrt_contigs-mgmt_unitig_0_quiver:c1:p1162706	unitig_1_quiver	85.00	20	3	0	1	20	33	61	37169	37188	41174	0.037	23.8
metacrt_contigs-mgmt_unitig_0_quiver:c1:p1162889	unitig_1_quiver	100.00	12	0	0	12	23	33	36	8686	8697	41174	0.34	20.6
metacrt_contigs-mgmt_unitig_0_quiver:c1:p1162950	unitig_1_quiver	84.21	19	3	0	15	33	33	85	24864	24882	41174	0.11	22.2
metacrt_contigs-mgmt_unitig_0_quiver:c1:p1162950	unitig_1_quiver	87.50	16	2	0	6	21	33	85	6536	6521	41174	0.34	20.6
metacrt_contigs-mgmt_unitig_0_quiver:c1:p1162950	unitig_1_quiver	80.00	20	4	0	13	32	33	85	12655	12636	41174	0.34	20.6
metacrt_contigs-mgmt_unitig_0_quiver:c1:p1163011	unitig_1_quiver	80.95	21	4	0	3	23	33	64	33159	33179	41174	0.11	22.2
metacrt_contigs-mgmt_unitig_0_quiver:c1:p1163072	unitig_1_quiver	83.33	18	3	0	4	21	33	55	28501	28518	41174	0.34	20.6
metacrt_contigs-mgmt_unitig_0_quiver:c1:p1163133	unitig_1_quiver	81.82	22	4	0	12	33	33	82	3166	3145	41174	0.037	23.8
metacrt_contigs-mgmt_unitig_0_quiver:c1:p1163133	unitig_1_quiver	84.21	19	3	0	7	25	33	82	9746	9728	41174	0.11	22.2
metacrt_contigs-mgmt_unitig_0_quiver:c1:p1163133	unitig_1_quiver	100.00	12	0	0	9	20	33	82	22598	22609	41174	0.34	20.6
metacrt_contigs-mgmt_unitig_0_quiver:c1:p1163194	unitig_1_quiver	93.33	15	1	0	17	31	33	48	29573	29587	41174	0.11	22.2
metacrt_contigs-mgmt_unitig_0_quiver:c1:p1163194	unitig_1_quiver	93.33	15	1	0	18	32	33	48	34061	34075	41174	0.11	22.2
metacrt_contigs-mgmt_unitig_0_quiver:c1:p1163255	unitig_1_quiver	85.00	20	3	0	1	20	33	82	10466	10485	41174	0.037	23.8
metacrt_contigs-mgmt_unitig_0_quiver:c1:p1163255	unitig_1_quiver	100.00	13	0	0	15	27	33	82	16513	16501	41174	0.11	22.2
metacrt_contigs-mgmt_unitig_0_quiver:c1:p1163255	unitig_1_quiver	92.86	14	1	0	14	27	33	82	28276	28289	41174	0.34	20.6

VIRSorter

https://github.com/simroux/VirSorter

Prediction of viral sequences in unitiq_0 and unitiq_1:

```
## 3 - Complete phage contigs - category 3 (not so sure)
## Contig_id,Nb genes contigs,Fragment,Nb genes,Category,Nb phage hallmark genes,Phage gene enrichment sig,Non-Caudovirales phage gene enrichment sig,Pfam depletion sig,U ncharacterized enrichment sig,Strand switch depletion sig,Short genes enrichment sig
VIRSorter_unitig_1_quiver,47,VIRSorter_unitig_1_quiver,47,3,,,,gene_10-gene_47:12.33803784004104,gene_10-gene_47:5.09649532164305,,

## 6 - Prophages - category 3 (not so sure)
## Contig_id,Nb genes contigs,Fragment,Nb genes,Category,Nb phage hallmark genes,Phage gene enrichment sig,Non-Caudovirales phage gene enrichment sig,Pfam depletion sig,U ncharacterized enrichment sig,Strand switch depletion sig,Short genes enrichment sig
VIRSorter_unitig_0_quiver,2517,VIRSorter_unitig_0_quiver-gene_1000-gene_1112,113,3,,,,gene_1000-gene_1099:27.39602990747906,gene_1056-gene_1112:5.88239235294574,,
```

Potential prophage sequence in unitiq_0: 1044425-1148207

Supplemental references

1. Darzentas N. 2010. Circoletto: Visualizing sequence similarity with Circos. Bioinformatics 26:2620–2621.

2. Cardenas JP, Lazcano M, Ossandon FJ, Corbett M, Holmes DS, Watkin E. 2014. Draft Genome Sequence of the Iron-Oxidizing Acidophile Leptospirillum ferriphilum Type Strain DSM 14647. Genome Announc 2:e01153-14-e01153-14.

3. Qi J, Wang B, Hao BI. 2004. Whole Proteome Prokaryote Phylogeny Without Sequence Alignment: A K-String Composition Approach. J Mol Evol 58:1–11.

4. Wang Y, Coleman-Derr D, Chen G, Gu YQ. 2015. OrthoVenn: A web server for genome wide comparison and annotation of orthologous clusters across multiple species. Nucleic Acids Res 43:W78–W84.
